# The Phylogenomic Framework and Infrageneric Classification of Temperate Asian Caraganeae (Leguminosae, Papilionoideae)

**DOI:** 10.1002/ece3.72638

**Published:** 2025-12-09

**Authors:** Na Wang, Pei‐Liang Liu, Ling Zhang, Rui Ma, Liang Zhao, Hui Wang, Zhao‐Yang Chang

**Affiliations:** ^1^ College of Life Sciences Northwest A&F University Yangling Shaanxi China; ^2^ Herbarium of Northwest A&F University (WUK) Yangling Shaanxi China; ^3^ Key Laboratory of Resource Biology and Biotechnology in Western China, Ministry of Education Northwest University Shaanxi China; ^4^ College of Life Sciences Tarim University Xinjiang China; ^5^ Key Laboratory of Biological Resource Protection and Utilization of Tarim Basin Xinjiang Production & Construction Group Xinjiang China

**Keywords:** *Calophaca*, *Caragana*, Fabaceae, *Halimodendron*, IRLC legumes, phylogeny, taxonomy

## Abstract

The legume tribe Caraganeae, consisting of the genera *Caragana*, *Calophaca*, and *Halimodendron*, is a mid‐sized, shrubby clade mainly found in temperate Central and Eastern Asia. Within this tribe, *Caragana* is the largest genus comprising 63–74 species, and it is recognized for its taxonomic complexity. However, the phylogenetic framework of Caraganeae and the infrageneric delimitations in *Caragana* remain inadequately resolved. In this study, fifty‐four species of Caraganeae were sampled, including 51 *Caragana* species representing all sections in previous taxonomical treatments. By using the genome skimming approach, we reconstructed the phylogenetic framework, inferred the infrageneric delimitations, and explored the historical diversification of the tribe. Our results demonstrated that Caraganeae is monophyletic, yet significant phylogenetic discordance in tree topology between the complete chloroplast genome and nuclear ribosomal DNA datasets was observed at both deep and shallow nodes. Within Caraganeae, the cp genome tree resolved nine highly supported lineages, with *Calophaca* and *Halimodendron* nested within *Caragana*. Molecular dating estimates suggest that Caraganeae originated during the Oligocene (stem age, 27.47 Ma, 95% HPD: 16.48–38.44 Ma), and diversified during the mid‐Miocene (crown age, 16.79 Ma, 95% HPD: 7–30.13 Ma). These findings provide important insights for clarifying the phylogenetic relationships in Caraganeae. The observed cytonuclear discordance may result from hybridization and/or incomplete lineage sorting. Furthermore, climatic and geological changes since the Eocene–Oligocene Transition—including global cooling, progressive aridity, and the rapid uplift of the Qinghai‐Tibetan Plateau—likely played essential roles in driving radiative diversification of Caraganeae.

## Graphical abstract

A phylogenomic framework of the temperate Asian tribe Caraganeae, and the revision to the infrageneric delimitations in the genus *Caragana* were proposed. Our results revealed that Caraganeae is monophyletic, but phylogenetic discordance in tree topology between the complete chloroplast genome and nuclear ribosomal DNA dataset were identified at both deep and shallow nodes. Within Caraganeae, nine highly supported lineages were resolved in the cp genome tree, with *Calophaca* and *Halimodendron* nested within *Caragana*.

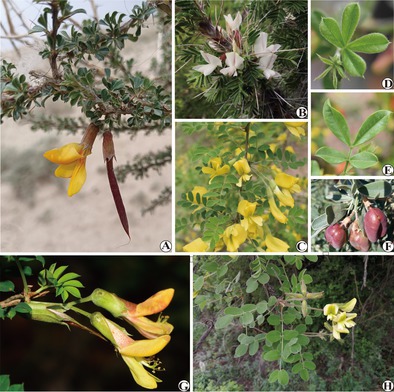

## 1 Introduction

The legume family (Leguminosae) is the third largest angiosperm family with over 22,708 species in approximately 809 genera, exhibiting remarkable species diversity and evolutionary success worldwide (Legume Phylogeny Working Group (LPWG) 2017; Bruneau et al. 2019). Within the largest subfamily, Papilionoideae, the “temperate herbaceous group” sensu Polhill (1981a, 1994) is well known for the vast radiation primarily in temperate regions of the Old World. Modern phylogenetic studies have revealed that most taxa in this group form a monophyletic clade, characterized by the loss of one copy of the 25‐kilobase inverted repeat (IR) in the chloroplast (cp) genome (Lavin et al. 1990; Wojciechowski et al. 2000). This clade, named as the IR‐lacking clade (“IRLC”), comprises more than 4000 species in about 56 genera, representing nearly one‐third of all papilionoid species (Wojciechowski et al. 2000; Duan et al. 2021).

Caraganeae Ranjbar, a mid‐sized tribe within the IRLC, was originally established by Ranjbar and Karamian (2003) based on the morphological similarities among five genera: *Caragana* Fabr., *Calophaca* Fisch. ex DC., *Halimodendron* Fisch. ex DC., *Chesneya* Lindl. ex Endl., and *Gueldenstaedtia* Fisch. Early molecular phylogenetic studies, primarily utilizing nuclear ribosomal (nr) ITS sequences, supported the monophyly of this group (Ahangarian et al. 2007; Amirahmadi et al. 2014; Ranjbar et al. 2015). Ranjbar et al. (2015) further classified the tribe into two subtribes: Caraganinae (*Caragana*, *Calophaca*, and *Halimodendron*) and Chesneyinae Ranjbar, F. Hajmoradi and Waycott (*Chesneya* and *Gueldenstaedtia*). However, when additional chloroplast DNA (cpDNA) markers (including both plastid genes and intergenic spacer regions) were applied, Duan et al. (2016) found that while each subtribe was monophyletic, Caraganeae as a whole was not. A more recent study employing cp genome and the complete nrDNA sequences reconstructed the phylogenetic framework of the IRLC legumes, and reclassified the monophyletic Caraganinae and Chesneyinae as separate tribes (Caraganeae and Chesneyeae, respectively; Duan et al. 2021). Consequently, our study defines Caraganeae as comprising three genera: *Caragana*, *Calophaca*, and *Halimodendron*. Caraganeae species are primarily distributed in temperate Central and Eastern Asia. Morphologically, they are characterized by having paripinnate leaves (except in *Calophaca*, which has imparipinnate leaves), a shrubby habit (rarely small trees), axillary flowers (typically solitary, geminate or fasciculate, rarely racemes), and non‐inflated legumes (except *Halimodendron*, which has inflated pods).

Historically, the placement of *Caragana*, *Calophaca*, and *Halimodendron* within the IRLC had been controversial, largely due to incongruent phylogenetic relationships reconstructed from cpDNA and nrDNA data (for a summary, see Table 1A and Figure S1). Early cpDNA marker studies by Wojciechowski et al. (2000) revealed a sister relationship between these genera and Hedysareae sensu Polhill (1981b). Lock (2005) followed Wojciechowski et al. (2000) and placed them within the tribe Hedysareae. Subsequent studies employing cp genome data yielded similar results (Duan et al. 2021). In contrast, nrDNA sequence analyses consistently showed these genera as sisters to Chesneyeae (Ahangarian et al. 2007; Amirahmadi et al. 2014; Ranjbar et al. 2015; Duan et al. 2021). As these studies were based on limited sequence regions or limited taxon sampling, the phylogenetic position of these genera in the IRLC, especially their relationship with Hedysareae and Chesneyeae, needs further investigation.

**TABLE 1:** Summary for the placement of Caraganeae in the IRLC (A), and the infrageneric classification of *Caragana* (B) in previous studies.

No.	Markers	Methods	Taxa no.	Caraganeae sampling^a^	Main results	References
**(A) The placement of Caraganeae**						
1	nrDNA: ITS	MP, ML	41	4	*Caragana*, *Calophaca*, and *Halimodendron* species formed a well‐supported clade, belonging to the Astragalean clade	Sanderson and Wojciechowski (1996)
2	cpDNA: *matK*, *rbcL*, *rpoC*	MRP	571	4	*Caragana*, *Calophaca*, and *Halimodendron* species formed a monophyletic group, belonging to the Hedysaroid clade	Wojciechowski et al. (2000)
3	Morphology	/	/	/	Established the tribe Caraganeae based on five genera: *Caragana*, *Calophaca*, *Halimodendron*, *Chesneya*, and *Gueldenstaedtia*	Ranjbar and Karamian (2003)
4	Literature review for taxonomic treatment	/	/	/	*Caragana*, *Calophaca*, and *Halimodendron* formed a clade, belonging to the tribe Hedysareae (following Wojciechowski et al. 2000)	Lock (2005)
5	nrDNA: ITS	MP	53	6	*Caragana*, *Calophaca*, *Halimodendron* species formed a well‐supported clade that was sister to a well‐supported clade composed of *Chesneya* and *Gueldenstaedtia*; the two clades formed a *Chesneya*‐*Caragana* clade (LBS = 66%) that was separated from either the Astragalean clade or the Hedysaroid clade	Ahangarian et al. (2007)
6	nrDNA: ITS; cpDNA: *matK*, *trnL*‐*F*	MP, ML, BI	44	2	*Caragana* and *Halimodendron* species formed a well‐supported clade that was sister to a well‐supported clade composed of *Chesneya* and *Gueldenstaedtia*; the two clades formed a so‐called Caraganean clade (PP = 1; LBS = 83%) that was separated from the tribe Hedysareae	Amirahmadi et al. (2014)
7	nrDNA: ITS	ML	56	14	Reintroduced Caraganeae (monophyletic, LBS = 72%), and it was classified into two subtribes: Caraganinae and Chesneyinae; Caraganinae was established based on three genera: *Calophaca*, *Caragana*, and *Halimodendron*	Ranjbar et al. (2015)
8	nrDNA: ITS; cpDNA: *matK*, *trnL*‐*F*, *psbA*‐*trnH*	ML; BI	97	39	Caraganeae sensu Ranjbar et al. (2015) is not recovered as monophyletic; support the monophyly of each of the two subtribes: Caraganinae (*Calophaca*, *Caragana*, and *Halimodendron*) and Chesneyinae	Duan et al. (2016)
9	nrDNA: 18S, ITS1, 5.8S, ITS2, and 26S; cp genome	ML; BI	114	4	Upgraded monophyletic Caraganinae (*Calophaca*, *Caragana*, and *Halimodendron*) and Chesneyinae to Caraganeae and Chesneyeae, respectively; Caraganeae was sister to Chesneyeae in the nrDNA tree (PP = 0.92, LBS = 97%), while it was sister to Hedysareae is the cpDNA tree (PP = 0.99, LBS = 79%)	Duan et al. (2021)
**(B) The infrageneric classification of *Caragana***						
1	Morphology	/	56	56	Three subgenera and five sections: subg. *Caragana* (sect. *Caragana*), subg. *Jubatae* Y. Z. Zhao [sect. *Jubatae* (Kom.) Y. Z. Zhao], subg. *Frutescentes* Y. Z. Zhao [sect. *Longispinae* Gorb., *Spinosae* (Kom.) Y. Z. Zhao, *Frutescentes* (Kom.) Sancz.]	Zhao (1993)
2	Morphology	/	72	72	Five sections: sect. *Caragana*, *Jubatae* (Kom.) Y. Z. Zhao, *Bracteolatae* (Kom.) M. L. Zhang, *Spinosae* (Kom.) Y. Z. Zhao, and *Frutescentes* (Kom.) Sancz.	Zhang (1997)^b^
3	nrDNA: ITS; cpDNA: *trnL*‐*F*, *trnS*‐*G*	MP, ML	21	20	Support the three subgenera and five sections sensu Zhao (1993), and each formed a monophyletic clade	Hou et al. (2008)
4	Morphology	/	63	63	Taxonomic revision based on the classification system of Zhao (1993)	Zhao (2009)
5	nrDNA: ITS; cpDNA: *rbcL*, *trnS*‐*G*	MP, ML, BI	52	50	Based on the classification system of Zhang (1997): both ITS and cpDNA data supported the monophyly of sect. *Caragana*, *Bracteolatae*, and *Frutescentes*; sect. *Jubatae* and *Spinosae* were not monophyletic	Zhang et al. (2009)
6	nrDNA: ITS; cpDNA: *matK*, *trnL*‐*F*, *psbA*‐*trnH*	ML, BI	97	39	Based on the classification system of Zhang (1997), supported the monophyly of six sections: sect. *Caragana*, *Bracteolatae*, *Spinosae*, *Frutescentes*, *Halimodendron* (Fisch. ex DC.) L. Duan, J. Wen & Zhao Y. Chang (newly designated section), and *Calophaca* (Fisch. ex DC.) L. Duan, J. Wen & Zhao Y. Chang (newly designated section); sect. *Jubatae* was not monophyletic	Duan et al. (2016)
7	nrDNA: ITS; cpDNA: *matK*, *rbcL*, *trnS*‐*G*, *atpB*‐*rbcL*, *psbA*‐*trnH*, *psbB*‐*N*	MP, ML, BI	71	64	Based on the classification system of Zhang (1997): six monophyletic groups were recognized as sect. *Caragana*, *Jubatae*, *Bracteolatae*, *Spinosae*, *Frutescentes* and *Tragacanthoides* (Pojark.) M. L. Zhang (newly designated section)	Zhang et al. (2016)^c^
8	Nuclear: genome‐wide SNPs and single‐copy genes; cp genome	ML	75	73	Based on the classification system of Zhao (2009), both nuclear and cpDNA data support the monophyly of six sections: sect. *Caragana*, *Halimodendron*, *Calophaca*, *Longispinae*, *Jubatae*, and *Frutescentes*; sect. *Spinosae* was not monophyletic in the cpDNA tree	Cui et al. (2025)

Abbreviations: BI, Bayesian inference; ML, maximum likelihood; MP, maximum parsimony; MRP, matrix representation with parsimony.

^a^ The tribe Caraganeae is confined to three genera: *Caragana*, *Calophaca*, and *Halimodendron*. See section “Introduction” for details.

^b^ The main difference between the classification system of Zhang (1997) and Zhao (1993): sect. *Bracteolatae* (Kom.) M. L. Zhang in Zhang (1997) largely equals sect. *Longispinae* Gorb. ser. *Bracteolatae* (Kom.) in Zhao (1993).

^c^ Newly designated sect. *Tragacanthoides* (Pojark.) M. L. Zhang comprises the species previously in sect. *Spinosae* (Kom.) Y. Z. Zhao and sect. *Jubatae* (Kom.) Y. Z. Zhao.

Within Caraganeae, *Caragana* is the largest genus and it has attracted much attention. Compared with about five to eight species in the genus *Calophaca* (Lock 2005; Zhu and Larsen 2010a; Zhang, Wen, et al. 2015) and only one species of *Halimodendron* (Zhu and Larsen 2010b), the genus *Caragana* comprises 63–74 species with most species occurring in China (Zhou 1996b; Zhang 1997; Zhao 2009). *Caragana* species often form the dominant components of the natural vegetation in arid and semi‐arid regions (Wu 1980; Zhang et al. 2009). It is also suggested that geoclimatic changes since the Tertiary, for example, global cooling and aridification at the Eocene–Oligocene Transition (EOT) and the Himalaya motion at mid‐Miocene, triggered and strongly affected the diversification of *Caragana* (Zhao 1993, 2009; Zhou 1996a; Zhou et al. 2005; Zhang et al. 2016). Therefore, it is regarded as a key taxon in understanding the origin and diversification of plants in temperate Asia.

**FIGURE 1:**
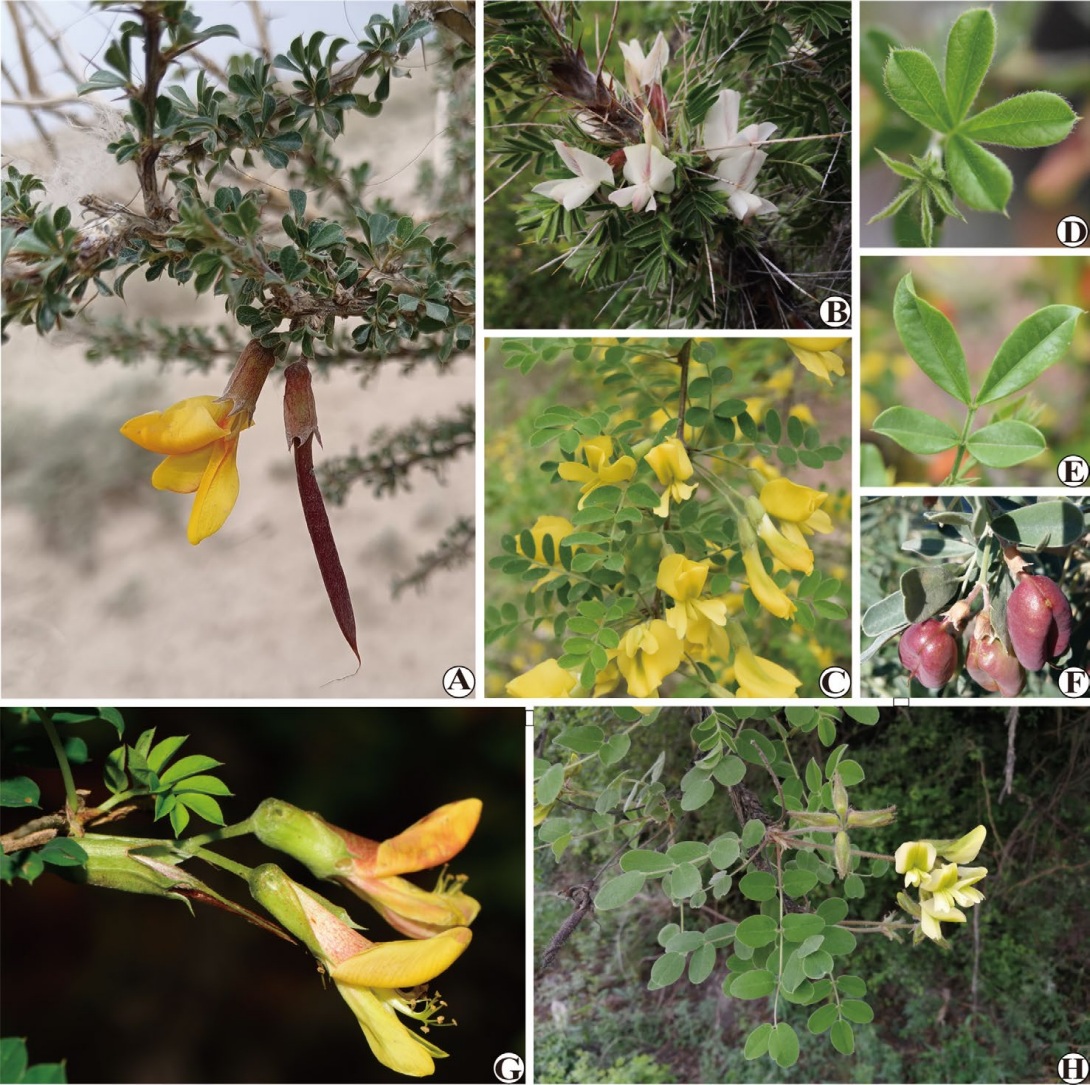
Habits and morphological diversity of representative species in Caraganeae. (A) *Caragana polourensis* showing typical arid and semi‐arid habits of Caraganeae; (B) *C. jubata* with solitary flowers, paripinnate leaves, and all rachises persistent; (C) *C. arborescens* with flowers 2–5 in a fascicle and all rachises caducous; (D, E) *C. rosea* and *C. sinica* with pseudopalmate leaves; (F) *Halimodendron halodendron* with 2–5‐flowered racemes; (G) *C. franchetiana* with flowers in pairs; (H) *Calophaca sinica* with 4–7‐flowered racemes and imparipinnate leaves. – Photo credits: A (Xinjiang, China) and B (Xinjiang, China) to Ling Zhang; C (Shaanxi, China), D (Shaanxi, China), and E (Shaanxi, China) to Jian Li; F (Xinjiang, China) to Zhao‐Yang Chang; G (Yunnan, China) to Xin‐Xin Zhu; H (Shanxi, China) to Pei‐Liang Liu.

The classification system of *Caragana* was fundamentally based on the work of Komarov (1908) and Pojarkova (1945), followed by Sanchir (1979, 1999), Gorbunova (1984), (Liu 1993, Liu et al. 2010), Zhao (1993, 2009), (Zhou 1996b, Zhou et al. 2005), and Zhang (1997). Because of high morphological variability both between and within species, as well as the potential hybrid origin of some species (e.g., *C. sinica* , Moore 1968; Zhou 1996b; Zhao 2009), the genus *Caragana* is taxonomically complex. Several important morphological characters are used for infrageneric delimitation, such as leaf rachis (caducous or persistent), leaf shape (paripinnate or pseudopalmate), number of leaflets (4, 6, or 6–20 foliolate), and the inflorescence (solitary, fasciculate, geminate, or umbellate with 3–4 flowers) (Figure 1). The micro‐morphological character of leaves, i.e., stomata presence on abaxial, adaxial, or both surfaces, is also suggested to be important (Chang 2008). Among the varying classification systems based on morphology, Zhao (1993, 2009) and Zhang (1997) have been subsequently supported or modified by molecular phylogenetic studies, with nrDNA ITS and cpDNA markers employed (Hou et al. 2008; Zhang et al. 2009, 2016; Duan et al. 2016; for details, see Table 1B). However, due to limited taxon sampling and/or insufficient sequence variation, these studies did not provide satisfactory resolution or support for infrageneric relationships. In a recent study, Cui et al. (2025) produced a chromosome‐level genome assembly for *C. arborescens* and resequenced 73 species to reconstruct the phylogeny of *Caragana*. Their findings emphasize the role of ancient hybridization in driving arid adaptation and species diversification. Despite this advance, their study did not thoroughly address infrageneric delimitation.

In this study, using the genome skimming approach (Straub et al. 2012; Zeng et al. 2018), we obtained cp genome and the complete nrDNA sequences from most of the currently recognized Caraganeae species in Central and Eastern Asia. Based on phylogenomic analyses, we aimed to (1) clarify the phylogenetic position of Caraganeae within the IRLC; (2) establish a broad phylogenetic framework of Caraganeae, especially focusing on infrageneric delimitations in *Caragana*; (3) explore the historical diversification of Caraganeae.

## 2 Materials and Methods

### 2.1 Taxon Sampling

**FIGURE 2:**
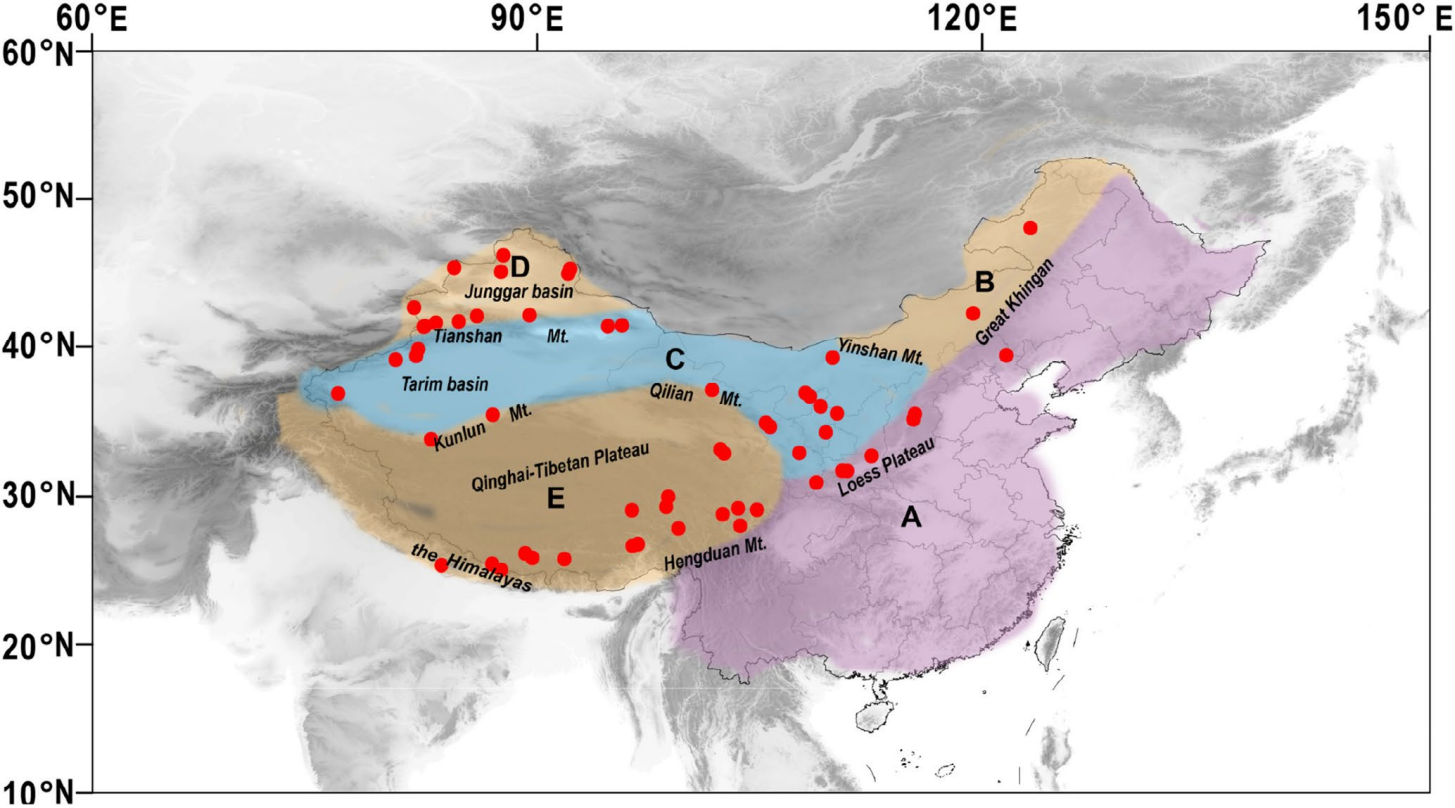
Map illustrates the distribution pattern of Caraganeae species sampled in China. The dots on the map indicate the collection sites where various Caraganeae species have been found, and distinct colors represent the different endemism regions (Zhang, Xue, et al. 2015). The distribution area is as follows: (A) Eastern Forest region; (B) Eastern Mongolia steppe region; (C) Kashgar and Inner Mongolia desert region; (D) Northern Xinjiang grassland region; (E) The Qinghai‐Tibetan Plateau.

We sampled 60 Caraganeae accessions comprising 51 species of *Caragana* Fabr., two of *Calophaca* Fisch. ex DC., and one of *Halimodendron* Fisch. ex DC. (Appendix A and Table S1). The sampling covered all major sections of *Caragana* proposed so far (Table 1B), and represented the taxonomic diversity from the broad geographic regions in China (Figure 2). For the genus *Caragana* s.s., we followed and accepted the taxonomy and classification of Zhang (1997). Additionally, we included four species of *Chesneya* Lindl. ex Endl. and one species of *Chesniella* Boriss. as representatives of closely related Chesneyeae taxa (Appendix A and Table S1). Voucher specimens were deposited in the herbarium of Northwest A&F University (WUK).

To establish a broader phylogenetic framework for placing Caraganeae in the IRLC and to estimate divergence times, we incorporated 31 species representing 24 other IRLC genera, covering all major IRLC tribes identified by Duan et al. (2021). *Robinia pseudoacacia* L. and *Sesbania cannabina* (Retz.) Poir. served as outgroups. The sequences of those species were downloaded from GenBank (see Appendix A).

### 2.2 DNA Isolation, Sequence Assembly, and Annotation

Total genomic DNA of 65 accessions representing 59 species was extracted from leaf samples preserved in silica gel or leaves of herbarium specimens with the DNAsecure Plant Kit (DP320; Tiangen Biotech, Beijing, China). DNA content and quality were assessed using a Qubit fluorometer (Invitrogen, Carlsbad, CA, USA) with the dsDNA HS kit, and also by visual assessment on 1% agarose gels. Blunt‐end DNA libraries were prepared using the NEBNext Ultra DNA Library Prep kit for Illumina (New England Biolabs, Ipswich, MA, USA) following the manufacturer's protocol. Indexed libraries were pooled in equimolar ratios and sequenced on an Illumina X Ten system (Illumina, San Diego, CA, USA) to generate 150 bp paired‐end reads. All library preparation and sequencing were performed by the Cloud Health Medical Group Ltd. (Shanghai, China). Raw sequence data have been deposited in the NCBI Sequence Read Archive (BioProject PRJNA998559).

The adaptor sequences and low‐quality reads were filtered using Fastp (Chen et al. 2018). Initially, the cp genomes were assembled using the de novo assembly program NOVOPlasty v2.6.1 (Dierckxsens et al. 2017) from high‐quality raw data, and the remaining samples were assembled using GetOrganelle v1.7.6.1 (Jin et al. 2020), with a K‐mer size of 39. Reference cp genomes included *Caragana microphylla* (GenBank accession no.: NC_032691.1), *Calophaca sinica* (GenBank accession no.: MN696543.1), and *Chesneya acaulis* (GenBank accession no.: MW053403.1). Both PGA (Qu et al. 2019) and GeSeq (Tillich et al. 2017) on the Chlorobox web server (https://chlorobox.mpimp‐golm.mpg.de) were conducted to annotate all the protein‐coding genes (CDSs), transfer RNA genes (tRNAs), and ribosomal RNA genes (rRNAs), with results between methods compared. Complete cp genomes were aligned using MAFFT v7 (Katoh and Standley 2013) and manually verified in Geneious v11.0.2 (Kearse et al. 2012). Start and stop codons and intron/extron boundaries for CDSs were also checked manually. Newly assembled plastomes were deposited in GenBank under accession numbers OQ999196–OQ999260.

For the complete nrDNA sequence assembly including ETS, 18S, ITS1, 5.8S, ITS2, and 26S ribosomal RNA genes, we used the nrDNA sequence of *Glycyrrhiza glabra* L. (GenBank accession no.: KX530459.1 for the 18S, ITS1, 5.8S, ITS2, and 26S regions) and *Halimodendron halodendron* (GenBank accession no.: JF409763 for the ETS region) as references. The nrDNA contigs were recognized by GetOrganelle, and contigs mapping to reference nrDNA were assembled by BLAST v2.12.0 (Beneventano et al. 2020). The nuclear ribosomal RNA genes and their boundaries with ITS regions were annotated in Geneious by comparison with the annotated references. The nrDNA sequences have been submitted to GenBank under accession numbers OR805182–OR805245 and OR813931.

### 2.3 Phylogenetic Analyses

Phylogenetic relationships were inferred from three datasets separately: the complete cp genomes, cp CDSs, and nrDNA. Each dataset was aligned using MAFFT implemented in Geneious with subsequent manual trimming. jModelTest v2.1.7 (Darriba et al. 2012) was used to choose the best‐fit nucleotide substitution model under the Bayesian Information Criterion (BIC). The GTR + F + G4 model was the most appropriate model for the whole cp genome, while the GTR + F + I + G4 model was selected for cp CDSs and nrDNA.

The Bayesian inference (BI; Rannala and Yang 1996; Mau et al. 1999) was conducted using MrBayes v3.2.5 (Ronquist and Huelsenbeck 2003; Ronquist et al. 2012). Each BI was performed by applying two independent runs of Markov chain Monte Carlo (MCMC) for 10,000,000 generations, and trees were sampled every 1000 generations (10,000 trees sampled in total). The first 2500 trees (25%) were discarded as burn‐in. Tracer v1.7 (Rambaut et al. 2018) was used to detect the convergence of MCMC chains, with the effective sample size (ESS) > 200. Maximum likelihood (ML) analyses were conducted using IQ‐TREE v1.6 (Nguyen et al. 2015) with the following settings: rapid bootstrap analysis with 1000 replicates followed by a search for the best‐scoring ML tree initiated from a random seed. Tree visualization was achieved in FigTree v1.4.3 (http://tree.bio.ed.ac.uk/software/figtree).

### 2.4 Divergence Time Estimation

Divergence times within Caraganeae were estimated using the dataset of complete cp genomes. During the alignment for cp genomes of the IRLC taxa, one of the two IR regions was removed for *Robinia pseudoacacia* and *Sesbania cannabina* . We applied an uncorrelated lognormal relaxed‐clock model in BEAST v2.5.2 (Bouckaert et al. 2019), and employed the nucleotide substitution model of GTR + G. Other parameters were left to default values. We constrained two node ages based on Duan et al. (2020): (1) the Hologalegina root at 50.6 Ma based on the results of Lavin et al. (2005) and (2) the Asian *Wisteria* stem at 17.0 Ma according to a reliable fossil record (Wang et al. 2006; He et al. 2011). Geological time scale nomenclature followed the International Commission on Stratigraphy (ICS: http://www.stratigraphy.org).

Parameter settings were established using BEAUti v2.5.2 (Bouckaert et al. 2019). We ran three independent MCMC analyses for 700,000,000 generations, sampling every 1000 generations, and discarding the first 25% as burn‐in. Tracer v1.7 (Rambaut et al. 2018) was used to verify whether ESS for all parameters was > 200. Trees from independent runs were combined in LogCombiner, and the maximum clade credibility (MCC) tree with mean heights was generated in TreeAnnotator. The MCC tree was annotated as a chronogram with mean ages for the nodes and 95% highest posterior density (HPD) intervals.

### 2.5 Ancestral Character Reconstruction

According to previous morphological studies and our observations, two important morphological characters used to delimit infrageneric taxa of *Caragana* were selected to trace their evolutionary history: (1) inflorescence and (2) leaf shape. Using the Bayesian tree generated from the dataset of complete cp genomes as a backbone, we reconstructed ancestral states using the Bayesian binary Markov chain Monte Carlo (BBM) method in RASP (Yu et al. 2020). Analyses ran for 50,000 generations with 9 hot chains and 1 cold chain, sampled every 100 generations from the cold chain, and applied the F81 + G model for changes among character states. Character states were coded as: (1) inflorescence: (A) one flower per peduncle (solitary or a few in a fascicle), (B) flowers in pairs, (C) umbel with 3–4 flowers, (D) raceme; (2) leaf shape: (A) pseudopalmate, (B) paripinnate, (C) paripinnate on long branchlets and pseudopalmate on short branchlets, (D) imparipinnate.

## 3 Results

### 3.1 Overview of the Chloroplast Genome and nrDNA Assembly

We successfully assembled cp genome sequences for all 65 Caraganeae and outgroup samples. The cp genomes of Caraganeae species ranged in size from 125,272 bp (*Caragana versicolor*_b) to 133,621 bp ( *C. altaica* ) (Appendix A and Table S1), each containing a single IR region. These plastomes exhibited GC contents between 34.2% and 35.2%, with highly conserved gene content and organization. Each genome contained 109–112 genes, comprising 75–77 CDSs, 29–31 tRNA genes, and four rRNA genes.

For nrDNA, we obtained complete assemblies for all 65 accessions, with Caraganeae sequence lengths ranging from 6124 bp ( *C. erinacea* ) to 6345 bp (*C. qingheensis*) (Appendix A and Table S1). Alignment of nrDNA sequences revealed 414 variable sites (6.93%), including 265 parsimony‐informative sites (4.44%).

### 3.2 Phylogenetic Relationships

**FIGURE 3:**
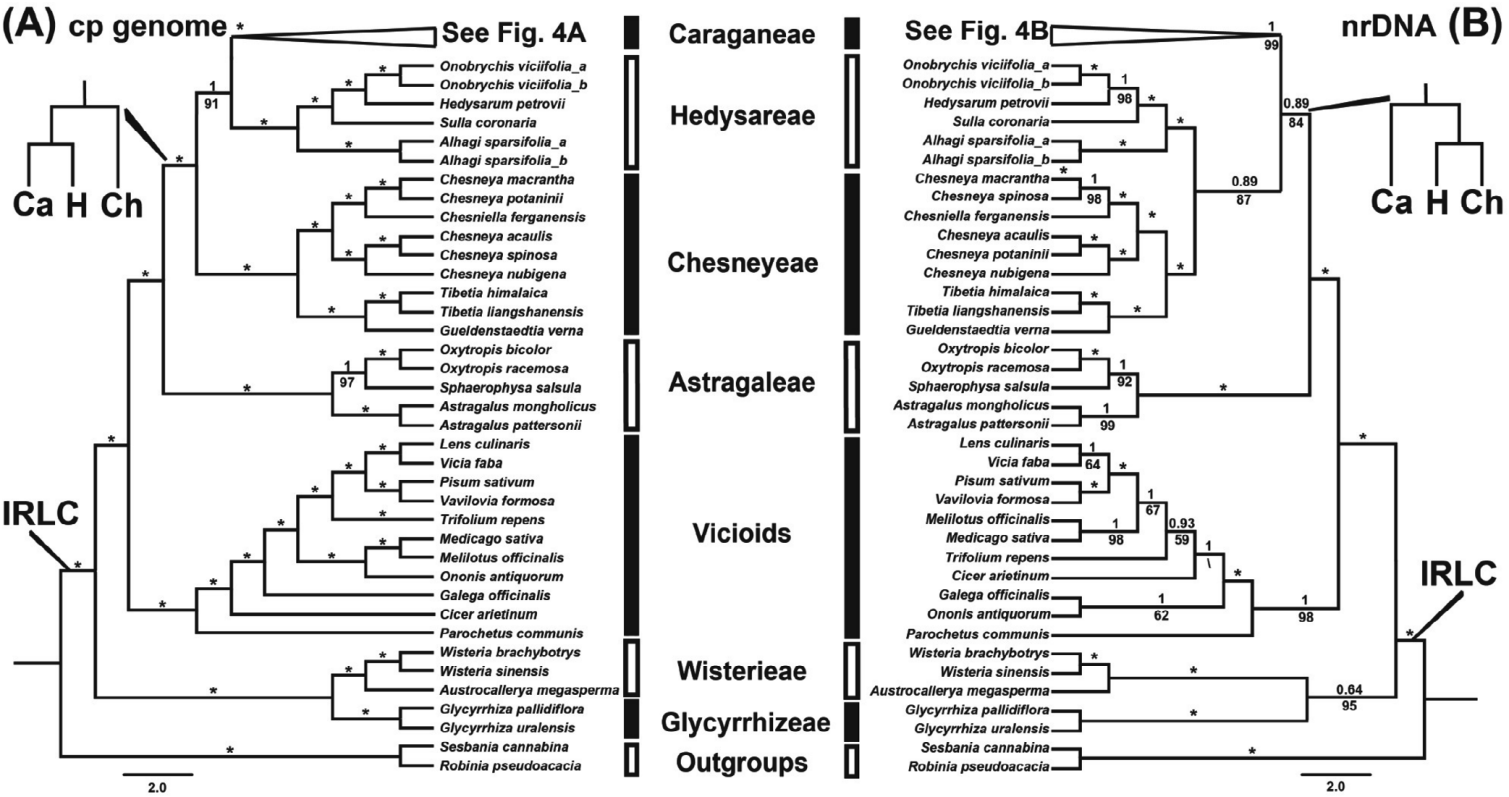
Bayesian maximum clade credibility trees of Caraganeae and its related taxa in IRLC, highlighting the phylogenetic position of Caraganeae. (A) The complete chloroplast genome dataset. (B) The nuclear ribosomal DNA dataset. Bayesian posterior probabilities are given above branches, and maximum likelihood bootstrap values are given below branches. Asterisks indicate PP = 1 and LBS = 100%.

**FIGURE 4:**
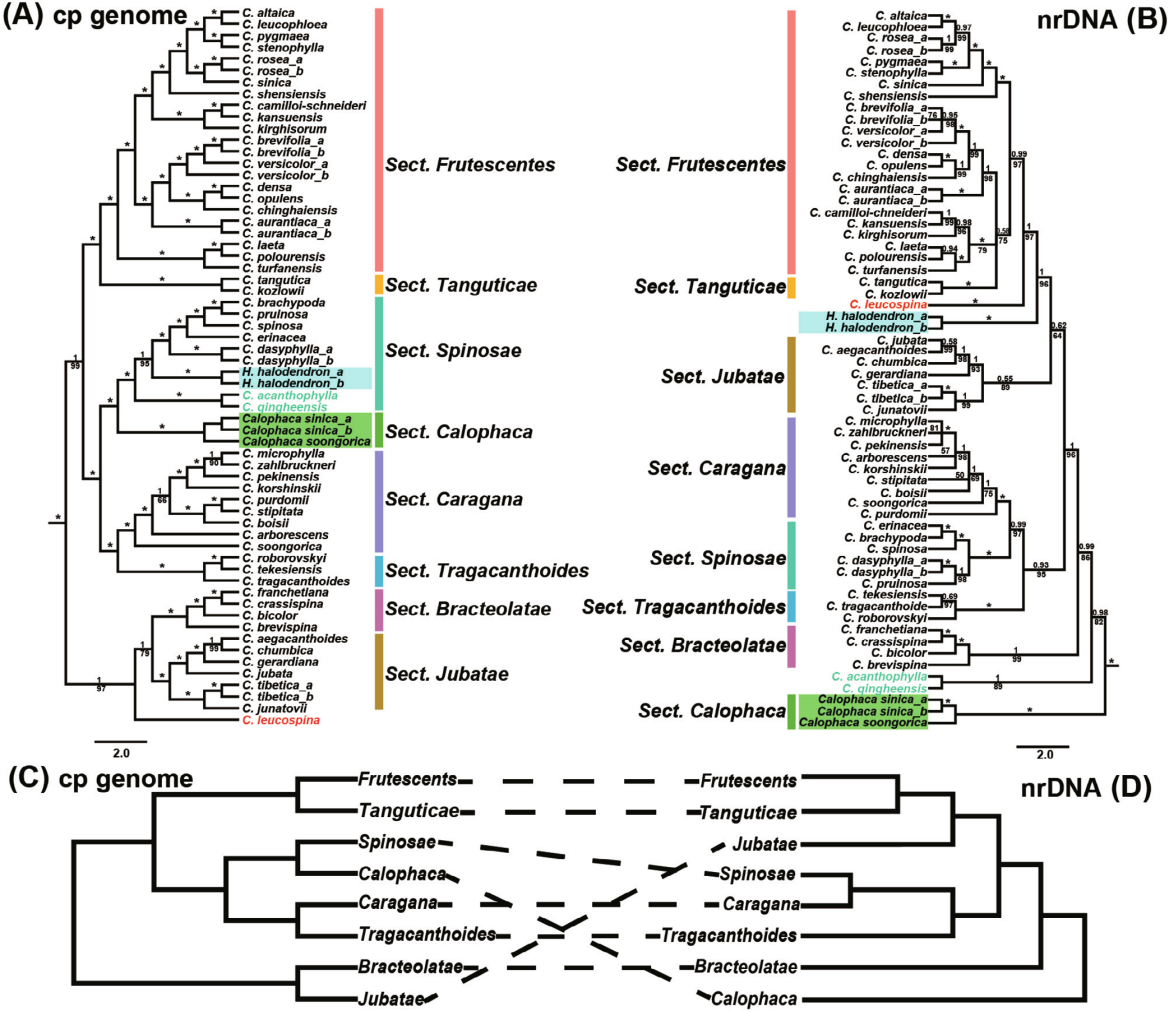
(A, B) Bayesian maximum clade credibility trees of the tribe Caraganeae, highlighting the phylogenetic structure within Caraganeae. (A) The complete chloroplast genome dataset. (B) The nuclear ribosomal DNA dataset. Bayesian posterior probabilities are given above branches, and maximum likelihood bootstrap values are given below branches. Asterisks indicate PP = 1 and LBS = 100%. (C, D) Summary of the incongruent topologies based on the cp genome dataset (left: C) and nrDNA dataset (right: D).

For the complete cp genome dataset, the BI analysis resulted in a well‐resolved tree (Posterior probabilities, PP = 1 for all branches), which was congruent in topology with the corresponding ML tree (Likelihood Bootstrap Support, LBS = 66%–100%) (Figures 3A and 4A). The concatenated dataset of cp CDSs resulted in a similar topology, differing in the relative position of sect. *Calophaca* within Caraganeae (Figures S2 and S3). As much higher support values were obtained using the complete cp genome dataset, we only discuss the phylogenetic tree based on this dataset.

Both cp genome and nrDNA data supported the monophyly of the tribe Caraganeae sensu Duan et al. (2021) (cp genome: PP = 1, LBS = 100%; nrDNA: PP = 1, LBS = 99%; Figure 3). However, the cp genome and nrDNA topologies showed incongruence. The cp genome topology showed Caraganeae and Hedysareae as monophyletic sister groups (PP = 1, LBS = 91%) that together formed a clade sister to Chesneyeae (PP = 1, LBS = 100%) (Figure 3A). In contrast, the nrDNA topology placed Chesneyeae and Hedysareae as sister groups (PP = 0.89, LBS = 87%) that together were sister to Caraganeae (PP = 0.89, LBS = 84%; Figure 3B).

Within Caraganeae, the cp genome data resolved nine strongly supported lineages (PP = 1, LBS = 100%) (Figure 4A). Six of these corresponded to previously recognized sections (Zhang et al. 2016): sect. *Frutescentes*, *Spinosae*, *Caragana*, *Tragacanthoides*, *Bracteolatae*, and *Jubatae*. We recovered sect. *Tanguticae* Sancz. sensu Sanchir (1999) as comprising *C. tangutica* and *C. kozlowii* (PP = 1, LBS = 100%). The former genus *Calophaca* was resolved as a distinct section within *Caragana* (sect. *Calophaca*). Notably, *C. leucospina* formed a separate lineage not assigned to any section. The nrDNA topology also recovered these eight sections plus *C. leucospina* as distinct lineages (PP = 0.55–1, LBS = 64%–100%; Figure 4B). Species composition within each section was almost the same as those in the cp genome tree, except that *Halimodendron halodendron* , *C. acanthophylla*, and *C. qingheensis* were separated from sect. *Spinosae*. Notably, at the section level, the cp genome and nrDNA topologies were largely incongruent (Figure 4C,D); in the nrDNA tree, sect. *Calophaca* was the sister group to the rest of *Caragana* (Figure 4D).

### 3.3 Divergence Time

**FIGURE 5:**
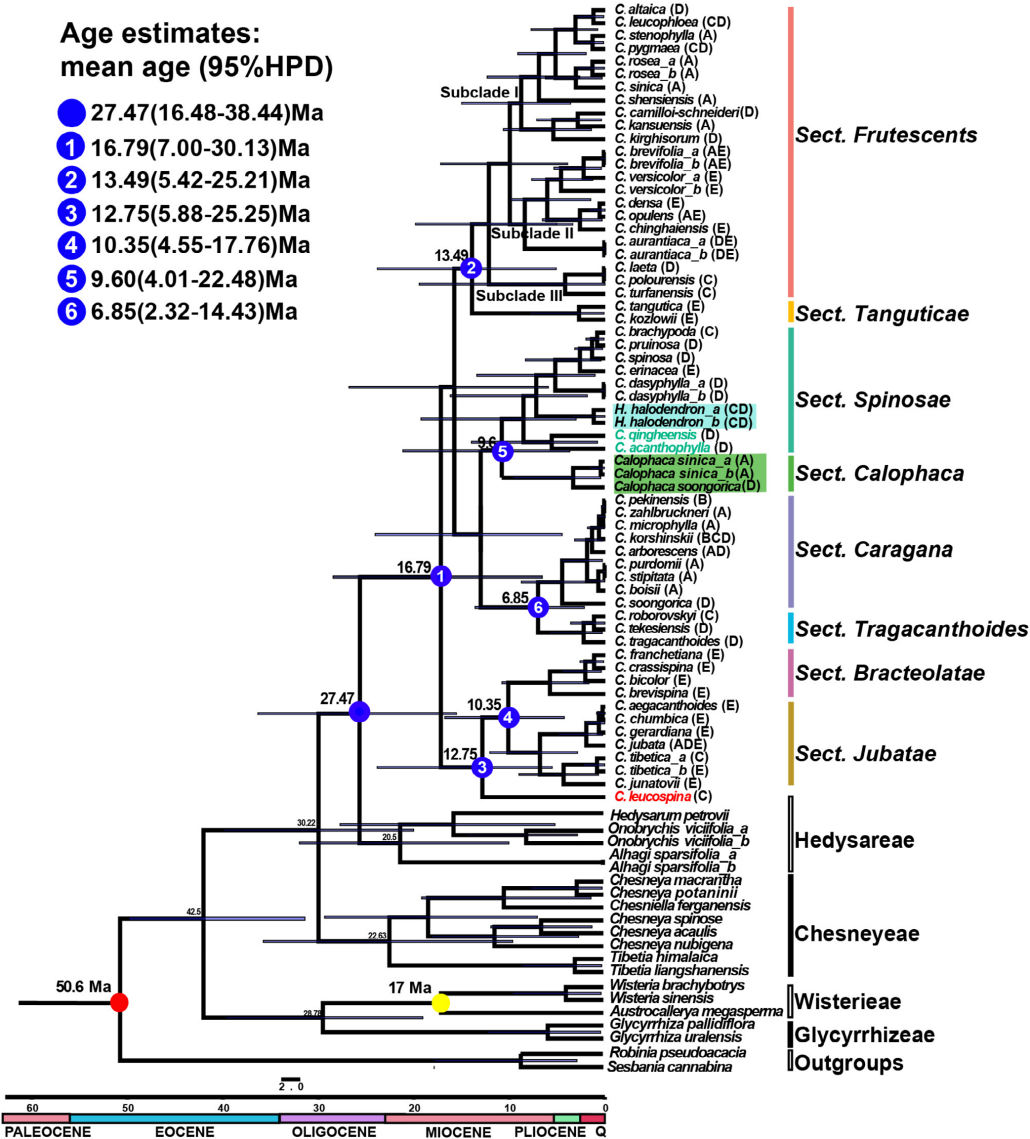
Chronogram showing divergence time of Caraganeae estimated in BEAST based on the complete chloroplast genome data. Blue bars represent 95% high posterior density for the estimated mean dates. For each Caraganeae species, Letters in the brackets represent distribution area in Figure 2.

Our molecular dating analysis (Figure 5) estimated that Caraganeae originated during the Oligocene at 27.47 Ma (stem age, node blue; 95% HPD: 16.48–38.44 Ma), and its crown age was dated to be at 16.79 Ma in the mid‐Miocene (node 1; 95% HPD: 7–30.13 Ma). The divergence of sect. *Tanguticae* from sect. *Frutescentes* was dated to be at 13.49 Ma (node 2; 95% HPD: 5.42–25.21 Ma). *Caragana leucospina* was inferred to have diverged from the species in sect. *Bracteolatae* and *Jubatae* at 12.75 Ma (node 3; 95% HPD: 5.88–25.25 Ma). Other major divergent events (nodes 4–6) are shown in Figure 5. The major lineages of Caraganeae appear to have radiated from the late Miocene through the Pleistocene.

### 3.4 Ancestral Character Reconstruction

The evolution of two key characters of Caraganeae is shown in suppl. Figure S4. Among them, one flower per peduncle (solitary or a few in a fascicle; probability of coding A: 99.88%) and paripinnate leaves (B: 88.94%) were reconstructed as the ancestral character types for the Caraganeae. For inflorescence type, the derived character states of flowers in pairs (B) and raceme (D) were inferred to originate twice, whereas an umbel with 3–4 flowers (C) originated once. For leaf shape, the derived character states of pseudopalmate leaves (A) and paripinnate on long branchlets and pseudopalmate on short branchlets (C) were inferred to originate twice, whereas imparipinnate leaves (D) originated once.

## 4 Discussion

### 4.1 On the Phylogenetic Position of Caraganeae

The monophyly of tribe Caraganeae sensu Duan et al. (2021) is well‐supported in our study, but the cp genome and nrDNA topologies are largely incongruent regarding the relationships among Caraganeae, Chesneyeae, and Hedysareae. Our cp genome data consistently place Caraganeae as sister to Hedysareae, aligning with previous studies either using cpDNA sequence regions (Wojciechowski et al. 2000) or complete cp genomes (Duan et al. 2021) (Figure S1A). When nrDNA sequences were employed, several studies reported a sister relationship of Caraganeae and Chesneyeae (ITS region: LBS = 66%, Ahangarian et al. 2007; PP = 1, LBS = 83%, Amirahmadi et al. 2014; LBS = 72%, Ranjbar et al. 2015; 18S, ITS1, 5.8S, ITS2, and 26S regions: PP = 0.92, LBS = 0.97%, Duan et al. 2021) (Figure S1B), while some studies reported poorly resolved relationships among these clades (ITS region: Sanderson and Wojciechowski 1996; Duan et al. 2016; Figure S1C). By incorporating ETS sequences and expanding the sampling of Chesneyeae species beyond Duan et al. (2021), our study provides a different scenario that Caraganeae is sister to the Chesneyeae + Hedysareae clade (PP = 0.89, LBS = 84%; Figure S1D). When the ETS region was removed from our dataset, the relationship is consistent with Duan et al. (2021) (PP = 0.99, LBS = 63%; Figure S5). While increased sequence data improve phylogenetic resolution, we are cautious that our nuclear sequences are still not sufficient to fully resolve their relationships.

Cytonuclear discordance is a fairly common phenomenon in plant phylogenetics, and various mechanisms have been proposed to explain it, such as allopolyploidy, paralogy of nrDNA, incomplete lineage sorting (ILS), and introgressive hybridization (specifically, “chloroplast capture”) (Rieseberg and Soltis 1991; Wendel and Doyle 1998). Allopolyploidy can probably be ruled out because most species of Caraganeae, Chesneyeae, and Hedysareae are diploids (2*n* = 16, rarely 2*n* = 14, e.g., the genus *Guldenstaedtia*; Ranjbar et al. 2015; Liu et al. 2017). Although nrDNA paralogy cannot be excluded, it is a less probable cause of the observed discordance. This is because nrDNA arrays within individuals are often homogeneous due to concerted evolution (Bailey et al. 2003). Distinguishing between ILS and hybridization can be challenging, especially in the context of ancient, rapid radiations when extensive ILS is plausible (reviewed in Stull et al. 2023). Genome‐wide nuclear data (e.g., low‐copy nuclear gene data) can be very helpful to disentangle the alternatives (e.g., Sun et al. 2015; Folk et al. 2017; Jin et al. 2025), and in our case, further studies incorporating such nuclear data are warranted.

### 4.2 Major Lineages Within Caraganeae

Our cp genome analysis clearly identified nine distinct lineages within Caraganeae, largely consistent with the clades reported by Zhang et al. (2016). Six sections were recovered in both studies: sect. *Frutescentes*, *Spinosae*, *Caragana*, *Tragacanthoides*, *Bracteolatae*, and *Jubatae*. The former genus *Calophaca* is recognized as a section embedded in *Caragana*. We additionally recovered sect. *Tanguticae* comprising *C. tangutica* and *C. kozlowii*, which form a sister relationship with sect. *Frutescentes*. A novel finding was the isolation of *C. leucospina* as a distinct lineage not assignable to any existing section. Despite some phylogenetic discordance in tree topology persisting at shallower nodes, the species composition within each section remained remarkably consistent between the cp genome and nrDNA trees, with the exception of three species in sect. *Spinosae*. This strong congruence supports the robustness of our infrageneric delimitations. The observed pattern suggests that potential hybridization and/or incomplete lineage sorting (ILS) events likely occurred either above the sectional level (representing ancient events) or below the sectional level, rather than between species from different sections. Indeed, with extensive nuclear data available, Cui et al. (2025) uncovered pervasive ancient hybridization, particularly between sections *Caragana* and *Calophaca*, which gave rise to other descendant lineages. Below, we outline the major lineages and discuss their taxonomic features.

#### 4.2.1 Lineage 1 (*C. leucospina*)

Previous morphological studies classified *C. leucospina* within sect. *Jubatae* (Zhao 1993, 2009). However, this species differs from other members of sect. *Jubatae* by its glaucous or white pubescent rachises, and its restricted distribution in the southern Tianshan Mountains (Xinjiang, Northwest China). Our study represents the first molecular phylogenetic analysis of this species, revealing it as a distinct lineage. Current data are insufficient to define a new section, but we recommend excluding it from sect. *Jubatae* pending further studies.

#### 4.2.2 Lineage 2 (Sect. *Jubatae*)

This section is characterized by persistent rachises and 6–14‐foliolate paripinnate leaves (Zhao 1993, 2009). Most species are distributed in East Himalaya and Hengduan Mountains, except for the more widely distributed *C. jubata* and *C. tibetica* (Figures 2, 5). Our sampling included seven accessions representing six taxa in this clade, and the only taxon which we failed to sample but was included in Zhang et al. (2016) is *C. changduensis*. Due to overlapping fruit trichome characteristics, Wu and Xia (1999) synonymized this species with *C. gerardiana*, which we sampled and have included in this section.

#### 4.2.3 Lineage 3 (Sect. *Bracteolatae*)

Distinguished by rachises persistent on long branchlets but caducous on short branchlets and 6–18‐foliolate paripinnate leaves, this section shows a distribution restricted to the Qinghai‐Tibetan Plateau (QTP) and Himalaya, similar to sect. *Jubatae*. Our cp genome tree supports the sister relationship between sect. *Bracteolatae* and sect. *Jubatae*, consistent with Zhang et al. (2016).

#### 4.2.4 Lineage 4 (Sect. *Tragacanthoides*)

The three species in this section were originally placed in either sect. *Jubatae* ( *C. roborovskyi* , *C. tekesiensis*) or sect. *Spinosae* (*C. tragacanthoides*) based on morphology (Zhao 1993, 2009; Zhang 1997). Zhang et al. (2016) first identified this as a distinct clade (i.e., sect. *Tragacanthoides*) using nrDNA ITS and six cpDNA markers, though without formal description. Our inclusion of previously unsampled *C. tekesiensis* supports its transfer from sect. *Jubatae*, and we formally describe this section in the “taxonomic treatment” section of this work. Species of this section are often found in mountain steppe/desert regions of northwest China, and it is regarded as a xerophytic group.

#### 4.2.5 Lineage 5 (Sect. *Caragana*)

Characterized by caducous rachises and 6–20‐foliolate paripinnate leaves, this group is well supported in previous molecular phylogenetic studies (Hou et al. 2008; Zhang et al. 2009, 2016; Duan et al. 2016), and our analyses confirmed the monophyly of the group. Distributed primarily in eastern Asia (eastern forest regions and mountain shrub steppe of northern China; Figures 2, 5), this mesophytic group was historically hypothesized as the most basally branching group of the genus *Caragana* (Moore 1968), and this perspective has been upheld by subsequent taxonomists (Sanchir 1974; Xu and Hao 1989; Zhao 1993, 2009; Zhou 1996a; Zhou et al. 2005; Zhang 1997). However, our study did not recover sect. *Caragana* as the basal group in either cp genome tree or nrDNA tree. Using extensive nuclear data, Cui et al. (2025) found that sect. *Caragana* is sister to a clade containing the remaining sections, which supports the traditional taxonomists' hypothesis; moreover, they also revealed that the presence of ILS is the main factor behind the phylogenetic discordance within section *Caragana*.

#### 4.2.6 Lineage 6 (Sect. *Calophaca*)

The embedded placement of *Calophaca* within *Caragana* in our cp genome tree is congruent with previous studies using cpDNA sequence regions (Duan et al. 2016; Zhang, Wen, et al. 2015; Zhang et al. 2016). Specifically, Zhang, Wen, et al. 2015, who sampled all of the eight currently described *Calophaca* species, strongly support the monophyly of this group (PP = 1, LBS = 96%). However, our nrDNA tree resolves *Calophaca* as the sister group to the rest of *Caragana*. Morphologically, *Calophaca* is characterized by imparipinnate leaves (vs. paripinnate in the genus *Caragana* s.s.), and racemes 4‐flowered or more (vs. solitary, fasciculate, geminate, or umbellate with 3–4 flowers in *Caragana* s.s.). Regarding distribution, only one species (*Calophaca sinica*, 2*n* = 16) is found in the eastern Asia forest region, where it was hypothesized to have early evolved (Chang et al. 2004). Most other species are disjunctively distributed in the mountain steppes of Central Asia, with one species extending westward to southeastern Europe and Russia. Given its distinct morphology, overlapping ancient distribution, and close phylogenetic relationship with the genus *Caragana* s.s., recent nuclear data propose that the common ancestor of all other sections likely originated within sect. *Calophaca* and *Caragana* through ancient hybridization (Cui et al. 2025).

#### 4.2.7 Lineage 7 (Sect. *Spinosae*)

This section features rachises persistent on long but caducous on short branchlets, with 4–6(8)‐foliolate pinnate leaves on long branchlets and 4‐foliolate digitate leaves on short ones. Species of the section occur in mountain steppe or desert regions of Central Asia (mainly Northwest China), and it is regarded as an xerophytic group. It is noted that *Halimodendron halodendron* nests within this group in the cp genome tree, which is congruent with previous studies using cpDNA sequence regions (Duan et al. 2016; Zhang et al. 2016). Morphologically, *Halimodendron* is distinct from the genus *Caragana* s.s. by its 2–5‐flowered racemes and inflated legumes, but it has characters similar to *Caragana* such as all rachises persistent and 2–4‐foliolate paripinnate leaves. As nrDNA also places *H. halodendron* within *Caragana*, we support Duan et al. (2016, 2021) in resurrecting the name *Caragana halodendron* (Pall.) Dum. Cours.

#### 4.2.8 Lineage 8 (Sect. *Tanguticae*)

**FIGURE 6:**
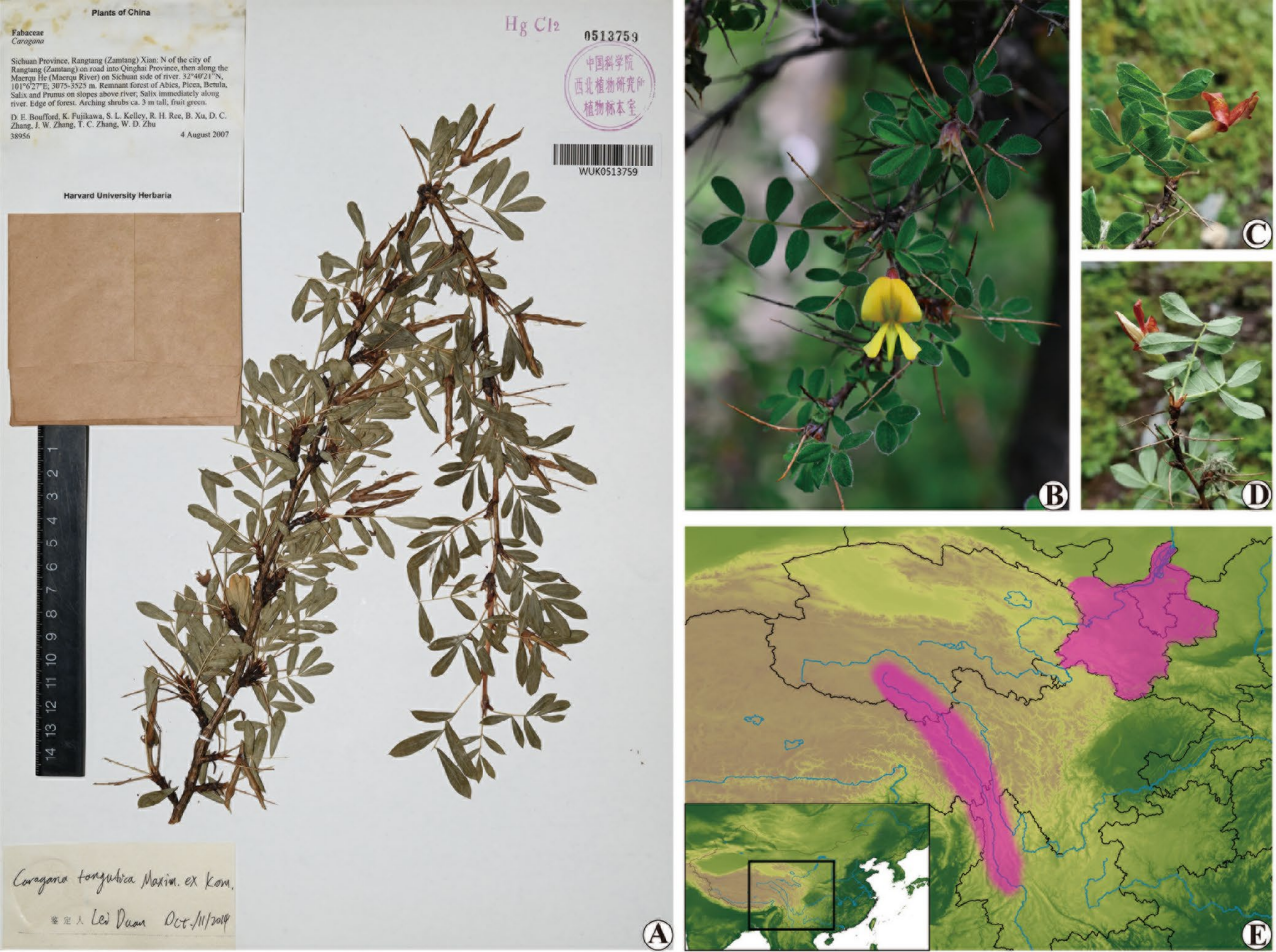
Sect. *Tanguticae* Sancz. Representative species: *Caragana Tangutica*. (A) The specimen photo; (B) Live plant photo; (C, D) Adaxial and abaxial views of leaves; (E) The distribution map (for details see Section 4.2.8 Sect. *Tanguticae*). Photo credits: B (Gansu, China) to Ren‐Bin Zhu; C (Gansu, China) and D (Gansu, China) to Jian‐Bin Pan.

The two species in this clade, i.e., *C. tangutica* and *C. kozlowii*, were originally assigned to sect. *Jubatae* for persistent rachises (Zhao 1993, 2009). However, molecular phylogenetic studies repeatedly recovered a closer relationship of the two species with sect. *Frutescentes* (Zhang et al. 2009, 2016; Duan et al. 2016; Cui et al. 2025). Our analysis is consistent with previous studies, and this clade is designated as sect. *Tanguticae*. Sect. *Tanguticae* was originally identified by Sanchir (1999) based on morphology, including four species: *C. chumbica*, *C. jubata* , *C. kozlowii*, and *C. tangutica* . We now confine this section to be composed of the latter two species, and the former two species are included in sect. *Jubatae* (Figure 4). Compared with other species in sect. *Jubatae*, species in this section have reduced leaflet numbers, usually (4)6–foliolate paripinnate leaves with enlarged apical pairs (Figure 6A–D). The distribution of this clade centers on the Lancang River Watershed, while *C. tangutica* extends to southern Gansu and Ningxia (Figure 6E).

#### 4.2.9 Lineage 9 (Sect. *Frutescentes*)

This section features rachises persistent on long but caducous short branchlets and 4‐foliolate pseudopalmate leaves. This is a well‐supported clade in previous studies using cpDNA sequence regions (Zhang et al. 2009, 2016; Duan et al. 2016), and our cp genome tree confirmed the monophyly of the section. This clade comprises 20–30 species and it is the largest clade in the genus *Caragana*. Our cp tree divides it into three subclades (Figure 5). Species in subclade I and III are mainly xeric, occurring in mountain steppe and/or desert regions of Central Asia (mainly Northwest China). Several species in subclade I dispersed into the eastern forest region (e.g., *C. sinica* , *C. rosea* , and *C. shensiensis*). Species of subclade II are mainly dispersed into the QTP and adapted to the cold, high‐altitude environments. Moreover, *C. sinica* has long been hypothesized to be a hybrid species (Moore 1968; Zhou 1996b; Zhao 2009). Our result suggests that *C. rosea* may have served as the putative paternal parent, and ancestral subclade I members may have been the putative maternal parent.

### 4.3 Divergence Times and Diversification of Caraganeae

Our study estimates that Caraganeae may have originated during the Oligocene (stem age, 27.47 Ma, 95% HPD: 16.48–38.44 Ma), and most of its clades diversified in the mid‐Miocene (crown age, 16.79 Ma, 95% HPD: 7–30.13 Ma). These estimates align with previous studies using cpDNA data. The first estimate for the stem age of Caraganeae was provided by Lavin et al. (2005), which was dated to be at 29.3 Ma (95% HPD, 21.1–35.4 Ma). Zhang et al. (2016) gave an estimation that Caraganeae was dated to 29.01 Ma (95% HPD, 28.04–29.99 Ma) for the stem age and 18.37 Ma (95% HPD, 12.11–25.87 Ma) for the crown age. Cui et al. (2025) also gave similar results (stem age, 29.06 Ma; crown age, 16.57 Ma). These divergence times correlate with significant geological and climatic events in Central Asia.

First, it is reported that global cooling and increased seasonal aridity at the EOT (EOT‐1 ca. 34.0 Ma, Oi‐1 ca. 33.5 Ma), had caused large‐scale and irreversible ecosystem changes in Central Asian steppe‐desert (reviewed in Barbolini et al. 2020). The origin and/or early diversification of the Caraganeae coincided with, or was probably promoted by the global/regional climatic or geological events at the EOT. Moreover, the Paleogene Central Tibetan Valley experienced ≥ 2 km of surface uplift between ~38 and 29 Ma, and by then a regionally contiguous proto‐plateau was established in Tibet (Xiong et al. 2022; Ding et al. 2022). We suspect that the uplift may have also contributed to triggering the origin of Caraganeae.

Second, geological evidence suggests that during the time period from ~25 Ma to ~15 Ma, the Himalaya Mountains in the south of QTP and Kunlun Mountains in the north were rapidly uplifted from < 3 km to near‐modern elevations (reviewed in Ding et al. 2022). It is possible that the rapid uplift of the Himalaya Mountains at ~20 Ma effectively blocked southerly moisture‐bearing winds, intensifying aridification northward. The crown‐node age of ca. 17 Ma falls within the range of the period of the Himalayan Motion, and we agree with Zhang et al. (2016) that the diversification of Caraganeae can be considered as the long‐term effects of arid climate driven by the Himalayan Motion. The Himalayan Motion and rapid uplift of QTP have also been suggested to accelerate the diversification of other lineages in IRLC legumes; for example, *Hedysarum* L. (crown age 16.92 Ma; Liu 2017), *Astragalus* L. (stem age 16.09 Ma, crown age 12.51 Ma; Su et al. 2021).

The subsequent diversification of nine main Caraganeae lineages occurred during the mid‐ to late Miocene (ca. 13.49–6.85 Ma). In Central Asia, the diversification in both the alpine biome and steppe‐desert lowlands during this time period appears to have been driven mainly by global cooling and progressive aridity (Barbolini et al. 2020). Moreover, the rapid uplift of the northern margin of the QTP (e.g., the uplift of Qilian Mountains and Tianshan Mountains) may generate orographic rain shadows, which could in turn modify precipitation patterns across relatively short distances and promote local species richness (Miao et al. 2012). During this time period, there could be two centers for species diversification in the genus *Caragana*: the north of Central Asia (Northern Xinjiang grassland region, D in Figure 2) and the QTP (E in Figure 2). In the QTP, species in sect. *Jubatae* and *Bracteolatae*, which kept ancestral paripinnate leaves while featuring persistent rachises and small/narrow leaves, were regarded as an adaptation to the cold and arid climate. In the Northern Xinjiang grassland region, species mainly in sect. *Spinosae* and *Frutescentes*, which were characterized by a reduced leaflet number (4–6 leaflets or even pseudopalmate leaves), were regarded as an adaptation to the arid climate. As per those xeric groups, sect. *Frutescentes* is the most successful group, with species dispersed into the eastern forest region and the QTP along the Hexi Corridor. Again, we recall that cytonuclear discordance in tree topology exists extensively at shallow nodes, suggesting that hybridization and/or ILS contribute to the diversification processes.

## 5 Taxonomic Treatment

### 5.1 *Caragana* sect. *Tragacanthoides* Na Wang, Hui Wang & Zhao Y. Chang ex. (Pojark.) M. L. Zhang, sect. nov. – Type: *Caragana tragacanthoides* (Pall.) Poir. (≡ *Robinia tragacanthoides* Pall.)

#### 5.1.1 Diagnosis and Note

Zhang et al. (2009, 2016) proposed the resurrection of ser. *Tragacanthoides* of Pojarkova (1945) at the sectional level. While Zhang et al. (2016) identified six potential members (*Caragana bongardiana*, *C. dasyphylla* , *C. pleiophylla*, *C. roborovskyi* , *C. tangutica* , and *C. tragacanthoides*) based on nrDNA ITS and six cpDNA markers, our cp genome and nrDNA analyses confirm the placement of *C. roborovskyi* , *C. tragacanthoides*, and the newly added *C. tekesiensis*. A recent phylogenomic analysis by Cui et al. (2025) supports the inclusion of *C. bongardiana* but excludes *C. pleiophylla*, resulting in four confirmed species of this section.

#### 5.1.2 Description

This section can be distinguished from section *Jubatae* by the following characters: leaflet blades narrowly elliptic to obovate, apex acute, and stomata on abaxial surfaces only (except for *C. tragacanthoides* with stomata on both adaxial and abaxial surfaces).

#### 5.1.3 Distribution and Habitat

Rocky, dry slopes; 700–3100 m. West of Nei Mongol, Northwest of Ningxia, Gansu, East of Qinghai, and Xinjiang in China; Kazakhstan, Kyrgyzstan, Uzbekistan, Mongolia, and Russia [temperate Central Asia].

Including four species, *Caragana roborovskyi* Kom., *C. tekesiensis* Y. Z. Zhao and D. W. Zhou, *C. bongardiana* (Fisch. and Mey.) Pojark., *C. Tragacanthoides* (Pall.) Poir.

### 5.2 *Caragana* sect. *Tanguticae* Sancz., op. cit. 30, 4 (1999) 507, p.p., excl. *Caragana chumbica* Prain, *C. jubata* (Pall.) Poir. – Type: *Caragana tangutica* Maxim. (≡ *Caragana leduensis* Y. Z. Zhao, Y. H. Wu & L. Q. Zhao)

#### 5.2.1 Description

This section differs from section *Jubatae*, which includes *C. chumbica* and *C. jubata* , by the following diagnostic characters: leaves typically 6‐foliolate (rarely 4), paripinnate; leaflets larger (8–15 × 3–8 mm) with the apical pair often the largest on the leaf; adaxial leaflet surfaces glabrous (lacking trichomes).

#### 5.2.2 Distribution and Habitat

River basins, slopes; 2000–4300 m. South of Qinghai, East of Xizang, West of Sichuan (Langcang River basin), south of Gansu, and Ningxia in China.

Including two species, *Caragana tangutica* Maxim., *C. kozlowii* Kom.

### 5.3 Key to the Eight Major Clades of Caraganeae

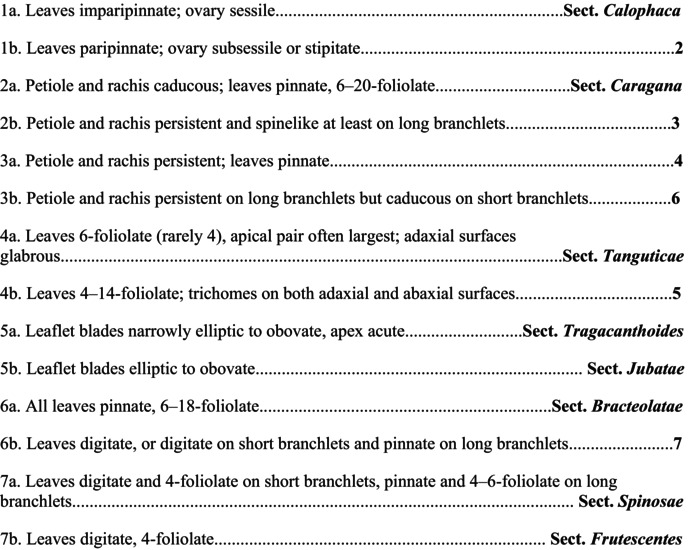

## Author Contributions

**Na Wang:** data curation (lead), formal analysis (lead), investigation (lead), methodology (lead), visualization (lead), writing – original draft (equal), writing – review and editing (supporting). **Pei‐Liang Liu:** conceptualization (supporting), data curation (supporting), formal analysis (supporting), methodology (supporting), writing – review and editing (supporting). **Ling Zhang:** formal analysis (supporting), methodology (supporting). **Rui Ma:** data curation (supporting), formal analysis (supporting). **Liang Zhao:** methodology (supporting), writing – review and editing (supporting). **Hui Wang:** conceptualization (supporting), formal analysis (supporting), funding acquisition (supporting), supervision (supporting), writing – original draft (equal), writing – review and editing (lead). **Zhao‐Yang Chang:** conceptualization (lead), funding acquisition (lead), investigation (supporting), resources (lead), supervision (lead), writing – review and editing (supporting).

## Funding

This work was supported by the Project of National Plant Specimen Resource Center of China, E0117G1001; The Science and Technology Basic Work of China, 2013FY112100; The foundation for Doctoral Returnees of Shaanxi Province of China, F2020221013; National Natural Science Foundation of China, 31110103911.

## Conflicts of Interest

The authors declare no conflicts of interest.

## Supporting information


**Data S1:** ece372638‐sup‐0001‐Supinfo01.docx.

## Data Availability

The raw sequence data used in this study were deposited in NCBI under the BioProject accession number PRJNA998559. The assembled chloroplast genome sequences have been submitted to the GenBank database under accession numbers OQ999196‐OQ999260. The nuclear ribosomal DNA sequences have been submitted to the GenBank database under accession numbers OR805182‐OR805245, and OR813931.

## Appendix A Accessions of Caraganeae and Outgroups Used in This Study

**Table:**

The list of GenBank accessions used in this study is presented as follows: Taxon, unique identifier, collector and collection number for the voucher (herbarium), collection locality, BioProject, SRA accession, GenBank accession number for chloroplast genome, chloroplast length (bp), GenBank accession number for nrDNA, nrDNA length (bp). A dash (−) indicates data that were unavailable. Sequences generated for this study are marked with an asterisk (*). The designations of a and b are used to discriminate different accessions of the same species.
***Caragana acanthophylla*** Kom., WN030, *Z.Y. Chang & al. 2019125* (WUK), China: Xinjiang, PRJNA998559, SRR25436135, OQ999196*, 130394, OR805184*, 6337; ***Caragana aegacanthoides*** (R. Parker) L. B. Chaudhary & S. K. Srivastava, WN019, *Z.Y. Chang & al. 2011357* (WUK), China: Xizang, PRJNA998559, SRR25436146, OQ999197*, 128420, OR805185*, 6339; ***Caragana altaica*** (Komarov) Pojarkova, WN005, *Z.Y. Chang & al. 2019243* (WUK), China: Xinjiang, PRJNA998559, SRR25436122, OQ999198*, 133621, OR805186*, 6338; ***Caragana arborescens*** Lam., WN057, *Z.Y. Chang & al. 2022003* (WUK), China: Shaanxi, PRJNA998559, SRR25436168, OQ999199*, 129397, OR805187*, 6341; ***Caragana aurantiaca*** Koehne, WN017, *Z.Y. Chang & al. 2019163* (WUK)^a^, China: Xinjiang, PRJNA998559, SRR25436148, OQ999200*, 133452, OR805188*, 6339; ***Caragana aurantiaca*** , WN020, *Z.Y. Chang & al. 2004292* (WUK)^b^, China: Xinjiang, PRJNA998559, SRR25436145, OQ999201*, 130539, OR805189*, 6339; ***Caragana bicolor*** Kom., WN055, *Z.Y. Chang & al. QZ‐211* (WUK), China: Sichuan, PRJNA998559, SRR25436170, OQ999202*, 129787, OR805190*, 6338; ***Caragana boisii*** C. K. Schneider, WN022, *Z.Y. Chang & al. QZ‐213* (WUK), China: Sichuan, PRJNA998559, SRR25436143, OQ999203*, 129397, OR813931*, 6340; ***Caragana brachypoda*** Pojark., WN010, *Z.Y. Chang & al. 2007087* (WUK), China: Gansu, PRJNA998559, SRR25436152, OQ999204*, 130291, OR805191*, 6340; ***Caragana brevifolia*** Kom., WN011, *Z.Y. Chang & al. 2007035* (WUK)^a^, China: Gansu, PRJNA998559, SRR25436179, OQ999205*, 130955, OR805192*, 6339; ***Caragana brevifolia***, WN068, *L.R. Xu & al. 96–010* (WUK)^b^, China: Gansu, PRJNA998559, SRR25436156, OQ999206*, 131917, OR805193*, 6339; ***Caragana brevispina*** Royle, WN050, *− 8350499* (KUN), Nepal, PRJNA998559, SRR25436176, OQ999207*, 130233, OR805194*, 6311; ***Caragana camilloi‐schneideri*** Kom., WN024, *Z.Y. Chang & al. 2004381* (WUK), China: Xinjiang, PRJNA998559, SRR25436141, OQ999208*, 131856, OR805195*, 6333; ***Caragana chinghaiensis*** Liou f., WN064, *Z.Y. Chang & al. 2010160* (WUK), China: Qinghai, PRJNA998559, SRR25436160, OQ999209*, 132556, OR805196*, 6339; ***Caragana chumbica*** Prain, WN023, *Z.Y. Chang & al. QZ‐673* (WUK), China: Xizang, PRJNA998559, SRR25436142, OQ999210*, 128441, OR805197*, 6339; ***Caragana crassispina*** Marq., WN066, *Z.Y. Chang & al. QZ‐584* (WUK), China: Xizang, PRJNA998559, SRR25436158, OQ999211*, 129694, OR805198*, 6339; ***Caragana dasyphylla*** Pojark., WN007, *Z.Y. Chang & al. 2019303* (WUK) ^a^, China: Xinjiang, PRJNA998559, SRR25436161, OQ999212*, 128957, OR805199*, 6340; ***Caragana dasyphylla***, WN029, *Z.Y. Chang & al. 2021019* (WUK)^b^, China: Xinjiang, PRJNA998559, SRR25436136, OQ999213*, 129174, OR805200*, 6338; ***Caragana densa*** Kom., WN026, *Z.Y. Chang & al. QZ‐312* (WUK), China: Sichuan, PRJNA998559, SRR25436139, OQ999214*, 130920, OR805201*, 6339; ***Caragana erinacea*** Kom., WN009, *Z.Y. Chang & al. QZ‐719* (WUK), China: Xizang, PRJNA998559, SRR25436154, OQ999215*, 129364, OR805202*, 6124; ***Caragana franchetiana*** Kom., WN033, *Z.Y. Chang & al. QZ‐606* (WUK), China: Xizang, PRJNA998559, SRR25436131, OQ999216*, 130453, OR805203*, 6339; ***Caragana gerardiana*** Royle, WN002, *Z.Y. Chang & al. QZ‐781* (WUK), China: Xizang, PRJNA998559, SRR25436180, OQ999217*, 127877, OR805204*, 6339; ***Caragana jubata*** (Pall.) Poir., WN004, *Z.Y. Chang & al. 2,019,150* (WUK), China: Xinjiang, PRJNA998559, SRR25436133, OQ999218*, 128210, OR805206*, 6339; ***Caragana junatovii*** Gorbunova, WN018, *Z.Y. Chang & al. 2010257* (WUK), China: Qinghai, PRJNA998559, SRR25436147, OQ999219*, 130542, OR805207*, 6339; ***Caragana kansuensis*** Pojark., WN041, *Y.P. Jin & Z.Y. Yu 1305* (WUK), China: Gansu, PRJNA998559, SRR25436123, OQ999220*, 131958, OR805208*, 6340; ***Caragana kirghisorum*** Pojark., WN061, *Z.Y. Chang & al. 2004219* (WUK), China: Xinjiang, PRJNA998559, SRR25436164, OQ999221*, 130075, OR805209*, 6334; ***Caragana korshinskii*** Kom., WN031, *Z.Y. Chang & al. 2019035* (WUK), China: Nei Mongol, PRJNA998559, SRR25436134, OQ999222*, 129696, OR805210*, 6341; ***Caragana kozlowii*** Kom., WN065, *Z.Y. Chang & al. QZ‐509* (WUK), China: Xizang, PRJNA998559, SRR25436159, OQ999223*, 130344, OR805211*, 6336; ***Caragana laeta*** Kom., WN035, *Z.Y. Chang & al. 2021093* (WUK), China: Xinjiang, PRJNA998559, SRR25436129, OQ999224*, 131450, OR805212*, 6340; ***Caragana leucophloea*** Pojark., WN058, *Z.Y. Chang & al. 2021049* (WUK), China: Xinjiang, PRJNA998559, SRR25436167, OQ999225*, 132886, OR805213*, 6338; ***Caragana leucospina*** Kom., WN006, *Z.Y. Chang & al. 2021049* (WUK), China: Xinjiang, PRJNA998559, SRR25436172, OQ999226*, 129827, OR805214*, 6303; ***Caragana microphylla*** Lam., WN053, *L.R. Xu 724* (WUK), China: Nei Mongol, PRJNA998559, SRR25436173, OQ999227*, 129240, OR805215*, 6341; ***Caragana opulens*** Kom., WN062, *Z.Y. Chang & al. QZ‐239* (WUK), China: Sichuan, PRJNA998559, SRR25436163, OQ999228*, 130953, OR805216*, 6339; ***Caragana pekinensis*** Kom., WN052, *F. Zhao, 271* (WUK), China: Hebei, PRJNA998559, SRR25436174, OQ999229*, 129353, OR805217*, 6341; ***Caragana polourensis*** Franch., WN008, *Z.Y. Chang & al. 2021065* (WUK), China: Xinjiang, PRJNA998559, SRR25436155, OQ999230*, 131695, OR805218*, 6340; ***Caragana pruinosa*** Kom., WN046, *Z.W. Zhang & al. 3381* (WUK), China: Xinjiang, PRJNA998559, SRR25436119, OQ999231*, 129646, OR805219*, 6340; ***Caragana purdomii*** Rehd., WN001, *Z.Y. Chang & al. 2010031* (WUK), China: Shaanxi, PRJNA998559, SRR25436181, OQ999232*, 129683, OR805220*, 6338; ***Caragana pygmaea*** (L.) DC., WN059, *− 78* (WUK), China: Nei Mongol, PRJNA998559, SRR25436166, OQ999233*, 132190, OR805221*, 6338; ***Caragana qingheensis*** Zhao Y. Chang, WN027, *Z.Y. Chang & al. 2019244* (WUK), China: Xinjiang, PRJNA998559, SRR25436138, OQ999234*, 128685, OR805222*, 6345; ***Caragana roborovskyi*** Kom., WN012, *Z.Y. Chang & al. 2019014* (WUK), China: Gansu, PRJNA998559, SRR25436178, OQ999235*, 129894, OR805223*, 6341; ***Caragana rosea*** Turcz. ex Maxim., WN013, *Z.Y. Chang & al. 2019002* (WUK)^a^, China: Shaanxi, PRJNA998559, SRR25436153, OQ999236*, 126155, OR805224*, 6338; ***Caragana rosea***, WN067, *Z.Y. Chang & al. 2022001* (WUK)^b^, China: Shaanxi, PRJNA998559, SRR25436157, OQ999237*, 125791, OR805225*, 6338; ***Caragana shensiensis*** C. W. Chang, WN063, *Z.Y. Zhang & C.S. Liu 17851* (WUK), China: Shaanxi, PRJNA998559, SRR25436162, OQ999238*, 130577, OR805226*, 6339; ***Caragana sinica*** (Buc'hoz) Rehd., WN054, *Z.Y. Chang & al. 2022002* (WUK), China: Shaanxi, PRJNA998559, SRR25436171, OQ999239*, 131696, OR805227*, 6340; ***Caragana soongorica*** Grub., WN016, *Z.Y. Chang & al. 2019153* (WUK), China: Xinjiang, PRJNA998559, SRR25436149, OQ999240*, 129082, OR805228*, 6340; ***Caragana spinosa*** (L.) DC., WN015, *Z.Y. Chang & al. 2004503* (WUK), China: Xinjiang, PRJNA998559, SRR25436150, OQ999241*, 129924, OR805229*, 6339; ***Caragana stenophylla*** Pojark., WN014, *Z.Y. Chang & al. 2019015* (WUK), China: Gansu, PRJNA998559, SRR25436151, OQ999242*, 131607, OR805230*, 6338; ***Caragana stipitata*** Kom., WN043, *− 2003.6.8* (WUK), China: Shaanxi, PRJNA998559, SRR25436121, OQ999243*, 129981, OR805231*, 6338; ***Caragana tangutica*** Maxim. ex Kom., WN056, Z.Y. *Chang & al. QZ‐315* (WUK), China: Sichuan, PRJNA998559, SRR25436169, OQ999244*, 130315, OR805232*, 6336; ***Caragana tekesiensis*** Y. Z. Zhao & D. W. Zhou, WN028, *Z.Y. Chang & al. 2019155* (WUK), China: Xinjiang, PRJNA998559, SRR25436137, OQ999245*, 129438, OR805233*, 6342; ***Caragana tibetica*** Kom., WN032, *Z.Y. Chang & al. 2010161* (WUK)^a^, China: Qinghai, PRJNA998559, SRR25436132, OQ999246*, 129378, OR805234*, 6338; ***Caragana tibetica***, WN047, *− 2004083* (WUK)^b^, China: Ningxia, PRJNA998559, SRR25436118, OQ999247*, 129085, OR805235*, 6338; ***Caragana tragacanthoides*** (Pall.) Poir., WN025, *Z.Y. Chang & al. 2019223* (WUK), China: Xinjiang, PRJNA998559, SRR25436140, OQ999248*, 129689, OR805236*, 6341; ***Caragana turfanensis*** (Krassn.) Kom., WN034, *Z.Y. Chang & al. 2021029* (WUK), China: Xinjiang, PRJNA998559, SRR25436130, OQ999249*, 130958, OR805237*, 6340; ***Caragana versicolor*** Benth., WN003, *Z.Y. Chang & al. 2010264* (WUK)^a^, China: Qinghai, PRJNA998559, SRR25436144, OQ999250*, 132415, OR805238*, 6337; ***Caragana versicolor***, WN060, *Z.Y. Chang & al. QZ‐395* (WUK)^b^, China: Sichuan, PRJNA998559, SRR25436165, OQ999251*, 125272, OR805239*, 6339; ***Caragana zahlbruckneri*** Schneid., WN051, *G.Y. Rao 1261* (WUK), China: Hebei, PRJNA998559, SRR25436175, OQ999252*, 129744, OR805240*, 6341; ***Calophaca sinica*** Rehd., WN036, *Z.Y. Chang & al. 2009157* (WUK)^b^, China: Shanxi, PRJNA998559, SRR25436128, OQ999253*, 130387, OR805182*, 6339; ***Calophaca soongorica*** Kar. et Kir., WN045, *L.R. Xu & al. 96–169* (WUK), China: Xinjiang, PRJNA998559, SRR25436120, OQ999254*, 129560, OR805183*, 6342; ***Halimodendron halodendron*** (Pall.) Voss., WN037, *Z.Y. Chang & al. 2019123* (WUK)^b^, China: Xinjiang, PRJNA998559, SRR25436127, OQ999255*, 129469, OR805205*, 6340; ***Chesniella ferganensis*** (Korshinsky) Borissova, WN049, *− 2004101* (WUK), China: Gansu, PRJNA998559, SRR25436177, OQ999256*, 129497, OR805245*, 6328; ***Chesneya macrantha*** S. H. Cheng ex H. C. Fu, WN038, *Z.Y. Chang & al. 2009054* (WUK), China: Xinjiang, PRJNA998559, SRR25436126, OQ999257*, 128716, OR805241*, 6327; ***Chesneya nubigena*** (D. Don) Ali, WN039, *Z.Y. Chang & al. 2011258* (WUK), China: Xizang, PRJNA998559, SRR25436125, OQ999258*, 129244, OR805242*, 6338; ***Chesneya potaninii*** (N. Ulziykh.) Govaerts, WN040, *Z.Y. Chang & al. 2019073* (WUK), China: Xinjiang, PRJNA998559, SRR25436124, OQ999259*, 129035, OR805243*, 6323; ***Chesneya spinosa*** P. C. Li, WN048, *Z.Y. Chang & al. 2011284* (WUK), China: Xizang, PRJNA998559, SRR25436117, OQ999260*, 126509, OR805244*, 6340; ***Calophaca sinica***, DL210, *P.L. Liu 394* (WUK)^a^, China: Shaanxi, PRJNA643162, SRR12136574, −, 129131, −, 6399; ***Halimodendron halodendron*** , DL209, *Z.Y. Chang & al. 2004370* (WUK)^a^, China: Xinjiang, PRJNA686216, SRR12136575, −, 129971, −, 6399; ***Alhagi sparsifolia*** Shap., DL214, *L. Duan 2016061* (IBSC)^a^, China: Xinjiang, PRJNA643162, SRR12136571, MT571455, 128233, −, 6410; ***Alhagi sparsifolia***, −, *T.G. Yang –* (−)^b^, China: Xinjiang, PRJNA685338, SRR13255665, ON550414, 128429, −, 6381; ***Hedysarum petrovii*** Yakovlev, DL216, *Z.Y. Chang & al. 2016050* (WUK), China: Nei Mongol, PRJNA643162, SRR12136570, MT120797, 122542, −, 6405; ***Onobrychis viciifolia*** Scop., DL257, *L. Duan 2018004* (IBSC)^a^, China: Ningxia, PRJNA706880, SRR13871747, MW007721, 121932, −, 6381; ***Onobrychis viciifolia*** , −, X. Fu – (−)^b^, China: Ningxia, PRJNA690644, SRR13385163, −, −, −, 6399; ***Sulla coronaria*** Medik., DL213, *P.L. Liu Duan2018005* (WUK), China: Shaanxi, PRJNA643162, SRR12136572, −, 122780, −, 6309; ***Chesneya acaulis*** (Baker) Popov., DL208, *Z.Y. Chang & al. 2013166* (WUK), China: Xizang, PRJNA643162, SRR12136576, −, 128629, −, 6399; ***Gueldenstaedtia verna*** (Georgi) Boriss., DL259, *Z.Y. Chang & al. 2003237* (WUK), China: Nei Mongol, PRJNA706880, SRR13871744, −, 122305, −, 6307; ***Tibetia himalaica*** (Baker) Tsui, DL258, *Z.Y. Chang & al. 2011085* (WUK), China: Xizang, PRJNA706880, SRR13871745, −, 123972, −, 6400; ***Tibetia liangshanensis*** P. C. Li, −, *Liuj 153027* (KUN), China: Sichuan, PRJNA725310, SRR14338556, MF193597, 123372, −, 6395; ***Astragalus mongholicus*** Bunge, −, *M. Jiang –* (−), China: Beijing, PRJNA330758, SRR3938254, −, −, −, 6385; ***Astragalus pattersonii*** A. Gray, −, *J.L.M. Charboneau 9805* (ARIZ), USA: Utah, PRJNA757771, SRR15673219, −, −, −, 6380; ***Sphaerophysa salsula*** (Pall.) DC., DL203, *P.L. Liu 2018149* (WNU), China: Shaanxi, PRJNA643162, SRR12136579, −, 123357, −, 6373; ***Oxytropis bicolor*** Bunge, −, *P.L. Liu 2018166* (WUK), China: Shaanxi, PRJNA643162, SRR12136585, MN255323, 122461, −, 6383; ***Oxytropis racemosa*** Turcz., DL206, *Z.Y. Chang & al. 2016097* (WUK), China: Nei Mongol, PRJNA643162, SRR12136577, −, 122310, −, 6384; ***Cicer arietinum*** L., −, *L. Duan 2020010* (IBSC), China: Guangdong, PRJNA706880, SRR13871741, −, 125162, −, 5827; ***Galega officinalis*** L., −, *L. Duan 2018014* (IBSC), China: Shaanxi, PRJNA643162, SRR12136581, −, 125152, −, 5736; ***Lens culinaris*** Medic., −, *L. Duan 2020009* (IBSC), China: Guangdong, PRJNA706880, SRR13871771, −, 123228, −, 6295; ***Medicago sativa*** L., LL01, *−*– (−), France, PRJNA255937, SRR25057510, −, −, −, 5802; ***Melilotus officinalis*** (L.) Pall., DL223, *L. Duan 2018008* (IBSC), China: Ningxia, PRJNA706880, SRR13871792, −, 127374, −, 6375; ***Ononis antiquorum*** L., −, *Z.W. Zhang et al. 4548* (WUK), China: Xinjiang, PRJNA706880, SRR13871777, −, 124776, −, 5992; ***Pisum sativum*** L., DL201, *L. Duan 2018013* (IBSC), China: Guangdong, PRJNA706880, SRR13871785, −, 122421, −, 6297; ***Parochetus communis*** Buch.‐Ham ex D. Don Prodr., DL226, *Z.Y. Chang & al. 2011140* (WUK), China: Xizang, PRJNA706880, SRR13871775, −, 123023, −, 6177; ***Trifolium repens*** L., −, *L. Duan 2018002* (IBSC), China: Shaanxi, PRJNA706880, SRR13871803, −, 132328, −, 6402; ***Vavilovia formosa*** (Steven) Fed., DL232, *A.L. Takhtajan & S.K. Czerepanov 4130* (US), Azerbaijan: Nakhchivan, PRJNA706880, SRR13871770, −, 121903, −, 6294; ***Vicia faba*** L., DL194, *L. Duan 2018010* (IBSC), China: Guangdong, PRJNA706880, SRR13871791, −, 123805, −, 5908; ***Austrocallerya megasperma*** (F. Muell.) J. Compton & Schrire, DL120, *J. Gillieatt 399* (K), Australia: Queensland, PRJNA706880, SRR13871746, −, 132418, −, 6422; ***Glycyrrhiza pallidiflora*** Maxim., DL113, *L. Duan 2016019* (IBSC), China: Xinjiang, PRJNA706880, SRR13871801, −, 127450, −, 6404; ***Glycyrrhiza uralensis*** Fisch., DL81, *Isherbay Sodombekov KPL‐00211* (MO), Kazakhstan: Issyk‐kul, PRJNA643162, SRR12136667, −, 127869, −, 6233; ***Wisteria brachybotrys*** Hemsl., DL161, *M.W. Chase 22664* (K), Japan: Niigata, PRJNA706880, SRR13871808, −, 131197, −, 6401; ***Wisteria sinensis*** (Sims) DC., DL118, *X.X. Su CSH15169* (CSH), China: Fujian, PRJNA643162, SRR12136584, −, 130927, −, 6402; ***Sesbania cannabina*** (Retz.) Poir., DL193, *L. Duan 2018009* (IBSC), China: Guangzhou, PRJNA643162, SRR12136582, −, 156702, −, 5750; ***Robinia pseudoacacia*** L., DL182, *L. Duan 2018003* (IBSC), China: Shaanxi, PRJNA643162, SRR12136583, −, 155531, −, 6413.
