# Supplementary material for: The Phylogenomic Framework and Infrageneric Classification of Temperate Asian Caraganeae (Leguminosae, Papilionoideae)
Source: Ecol Evol. 2025 Dec 9;15(12):e72638. doi: 10.1002/ece3.72638 (PMC12690163; doi:10.1002/ece3.72638)

**Supporting Information**

**Table S1.** Characteristics of chloroplast (cp) genomes and nuclear ribosomal DNA (nrDNA), and BioSample Accession numbers of sampled species in Caraganeae (1-60) and closely related Chesneyeae (61-65).

| **No.** | **Species** | **cp genome** | | |  | **nrDNA** | | **BioSample Accession** |
| --- | --- | --- | --- | --- | --- | --- | --- | --- |
|  |  | **Sequence length**  **(bp)** | **GC content**  **(%)** | **Number of genes**  **(CDS/tRNA/rRNA)** |  | **Sequence length**  **(bp)** | **GC content**  **(%)** |  |
| 1 | *Caragana acanthophylla* | 130394 | 34.4 | 111(76/31/4) |  | 6337 | 53.2 | SAMN36684630 |
| 2 | *Caragana aegacanthoides* | 128420 | 34.4 | 110(76/30/4) |  | 6339 | 53.1 | SAMN36684620 |
| 3 | *Caragana altaica* | 133621 | 34.9 | 111(76/31/4) |  | 6338 | 53.0 | SAMN36684606 |
| 4 | *Caragana arborescens* | 129397 | 34.4 | 111(76/31/4) |  | 6341 | 53.0 | SAMN36684655 |
| 5 | *Caragana aurantiaca_*a | 133452 | 34.4 | 111(76/31/4) |  | 6339 | 53.2 | SAMN36684618 |
| 6 | *Caragana aurantiaca_*b | 130539 | 34.6 | 111(76/31/4) |  | 6339 | 53.2 | SAMN36684621 |
| 7 | *Caragana bicolor* | 129787 | 35.2 | 111(76/31/4) |  | 6338 | 53.3 | SAMN36684653 |
| 8 | *Caragana boisii* | 129397 | 34.4 | 111(76/31/4) |  | 6340 | 53.1 | SAMN36684622 |
| 9 | *Caragana brachypoda* | 130291 | 34.6 | 111(76/31/4) |  | 6340 | 53.3 | SAMN36684611 |
| 10 | *Caragana brevifolia_*a | 130955 | 35.0 | 112(77/31/4) |  | 6339 | 53.2 | SAMN36684612 |
| 11 | *Caragana brevifolia_*b | 131917 | 34.9 | 112(77/31/4) |  | 6339 | 53.2 | SAMN36684666 |
| 12 | *Caragana brevispina* | 130233 | 34.6 | 111(76/31/4) |  | 6311 | 53.0 | SAMN36684648 |
| 13 | *Caragana camilloi-schneideri* | 131856 | 34.9 | 111(76/31/4) |  | 6333 | 53.2 | SAMN36684624 |
| 14 | *Caragana chinghaiensis* | 132556 | 34.7 | 111(76/31/4) |  | 6339 | 53.2 | SAMN36684662 |
| 15 | *Caragana chumbica* | 128441 | 34.4 | 110(76/30/4) |  | 6339 | 53.1 | SAMN36684623 |
| 16 | *Caragana crassispina* | 129694 | 35.1 | 111(76/31/4) |  | 6339 | 53.2 | SAMN36684664 |
| 17 | *Caragana dasyphylla_*a | 128957 | 34.5 | 111(76/31/4) |  | 6340 | 53.2 | SAMN36684608 |
| 18 | *Caragana dasyphylla_*b | 129174 | 34.6 | 111(76/31/4) |  | 6338 | 53.2 | SAMN36684629 |
| 19 | *Caragana densa* | 130920 | 34.8 | 110(75/31/4) |  | 6339 | 53.2 | SAMN36684626 |
| 20 | *Caragana erinacea* | 129364 | 34.7 | 111(76/31/4) |  | 6124 | 53.1 | SAMN36684610 |
| 21 | *Caragana franchetiana* | 130453 | 35.1 | 112(77/31/4) |  | 6339 | 53.2 | SAMN36684633 |
| 22 | *Caragana gerardiana* | 127877 | 34.5 | 110(76/30/4) |  | 6339 | 53.2 | SAMN36684603 |
| 23 | *Caragana jubata* | 128210 | 34.4 | 110(76/30/4) |  | 6339 | 53.1 | SAMN36684605 |
| 24 | *Caragana junatovii* | 130542 | 34.5 | 110(76/30/4) |  | 6339 | 53.2 | SAMN36684619 |
| 25 | *Caragana kansuensis* | 131958 | 34.7 | 111(76/31/4) |  | 6340 | 53.1 | SAMN36684641 |
| 26 | *Caragana kirghisorum* | 130075 | 35.0 | 111(76/31/4) |  | 6334 | 53.1 | SAMN36684659 |
| 27 | *Caragana korshinskii* | 129696 | 34.3 | 111(76/31/4) |  | 6341 | 53.1 | SAMN36684631 |
| 28 | *Caragana kozlowii* | 130344 | 34.6 | 111(76/31/4) |  | 6336 | 53.0 | SAMN36684663 |
| 29 | *Caragana laeta* | 131450 | 34.7 | 111(76/31/4) |  | 6340 | 52.9 | SAMN36684635 |
| 30 | *Caragana leucophloea* | 132886 | 34.9 | 110(76/30/4) |  | 6338 | 53.0 | SAMN36684656 |
| 31 | *Caragana leucospina* | 129827 | 34.4 | 111(76/31/4) |  | 6303 | 52.9 | SAMN36684607 |
| 32 | *Caragana microphylla* | 129240 | 34.3 | 111(76/31/4) |  | 6341 | 53.1 | SAMN36684651 |
| 33 | *Caragana opulens* | 130953 | 34.7 | 111(76/31/4) |  | 6339 | 53.2 | SAMN36684660 |
| 34 | *Caragana pekinensis* | 129353 | 34.3 | 111(76/31/4) |  | 6341 | 53.1 | SAMN36684650 |
| 35 | *Caragana polourensis* | 131695 | 34.7 | 111(76/31/4) |  | 6340 | 53.0 | SAMN36684609 |
| 36 | *Caragana pruinosa* | 129646 | 34.7 | 111(76/31/4) |  | 6340 | 53.1 | SAMN36684644 |
| 37 | *Caragana purdomii* | 129683 | 34.4 | 111(76/31/4) |  | 6338 | 53.0 | SAMN36684602 |
| 38 | *Caragana pygmaea* | 132190 | 34.8 | 111(76/31/4) |  | 6338 | 53.1 | SAMN36684657 |
| 39 | *Caragana qingheensis* | 128685 | 34.5 | 111(76/31/4) |  | 6345 | 53.1 | SAMN36684627 |
| 40 | *Caragana roborovskyi* | 129894 | 34.4 | 112(76/31/4) |  | 6341 | 53.1 | SAMN36684613 |
| 41 | *Caragana rosea_*a | 126155 | 35.0 | 109(76/29/4) |  | 6338 | 53.1 | SAMN36684614 |
| 42 | *Caragana rosea_*b | 125791 | 35.0 | 109(76/29/4) |  | 6338 | 53.1 | SAMN36684665 |
| 43 | *Caragana shensiensis* | 130577 | 34.6 | 110(76/30/4) |  | 6339 | 53.0 | SAMN36684661 |
| 44 | *Caragana sinica* | 131696 | 35.0 | 111(76/31/4) |  | 6340 | 53.0 | SAMN36684652 |
| 45 | *Caragana soongorica* | 129082 | 34.2 | 110(76/30/4) |  | 6340 | 53.2 | SAMN36684617 |
| 46 | *Caragana spinosa* | 129924 | 34.6 | 111(76/31/4) |  | 6339 | 53.3 | SAMN36684616 |
| 47 | *Caragana stenophylla* | 131607 | 34.8 | 111(76/30/4) |  | 6338 | 53.0 | SAMN36684615 |
| 48 | *Caragana stipitata* | 129981 | 34.3 | 111(76/31/4) |  | 6338 | 53.1 | SAMN36684642 |
| 49 | *Caragana tangutica* | 130315 | 34.7 | 111(76/31/4) |  | 6336 | 53.1 | SAMN36684654 |
| 50 | *Caragana tekesiensis* | 129438 | 34.5 | 111(76/31/4) |  | 6342 | 53.1 | SAMN36684628 |
| 51 | *Caragana tibetica_*a | 129378 | 34.6 | 110(76/30/4) |  | 6338 | 53.0 | SAMN36684632 |
| 52 | *Caragana tibetica_*b | 129085 | 34.6 | 110(76/30/4) |  | 6338 | 53.0 | SAMN36684645 |
| 53 | *Caragana tragacanthoides* | 129689 | 34.5 | 111(76/31/4) |  | 6341 | 53.2 | SAMN36684625 |
| 54 | *Caragana turfanensis* | 130958 | 34.7 | 111(76/31/4) |  | 6340 | 52.9 | SAMN36684634 |
| 55 | *Caragana versicolor_*a | 132415 | 34.9 | 111(76/31/4) |  | 6337 | 53.3 | SAMN36684604 |
| 56 | *Caragana versicolor_*b | 125272 | 35.0 | 109(76/29/4) |  | 6339 | 53.3 | SAMN36684658 |
| 57 | *Caragana zahlbruckneri* | 129744 | 34.3 | 111(76/31/4) |  | 6341 | 53.1 | SAMN36684649 |
| 58 | *Calophaca sinica*_b | 130387 | 34.6 | 111(76/31/4) |  | 6339 | 52.9 | SAMN36684636 |
| 59 | *Calophaca soongorica* | 129560 | 34.6 | 111(76/31/4) |  | 6342 | 52.8 | SAMN36684643 |
| 60 | *Halimodendron halodendron*_b^1^ | 129469 | 34.5 | 111(76/31/4) |  | 6340 | 53.0 | SAMN36684637 |
| 61 | *Chesniella ferganensis* | 129497 | 34.4 | 110(75/30/4) |  | 6328 | 53.4 | SAMN36684647 |
| 62 | *Chesneya macrantha* | 128716 | 34.3 | 110(76/30/4) |  | 6327 | 53.4 | SAMN36684638 |
| 63 | *Chesneya nubigena* | 129244 | 34.3 | 111(76/31/4) |  | 6338 | 53.0 | SAMN36684639 |
| 64 | *Chesneya potaninii* | 129035 | 34.3 | 110(76/30/4) |  | 6323 | 53.5 | SAMN36684640 |
| 65 | *Chesneya spinosa* | 126509 | 34.4 | 109(76/29/4) |  | 6340 | 53.3 | SAMN36684646 |

^1^ ***Halimodendron halodendron*** (Pallas) Druce, Rep. Bot. Soc. Exch. Club Brit. Isles 4: 626. 1917, also known as ***Caragana halodendron*** (Pallas) Dumont de Courset, Bot. Cult. 3: 513. 1802.

**Figure S1.** Phylogenetic relationships of Caraganeae, Chesneyeae, and Hedysareae. **A,** Caraganeae is sister to Hedysareae based on chloroplast (cp) DNA sequences (Wojciechowski et al., 2000; Duan et al., 2016; Duan et al., 2021); **B,** Caraganeae is sister to Chesneyeae based on nuclear ribosomal (nr) DNA sequences, i.e., ITS region (Ahangarian et al., 2007; Amirahmadi et al., 2014; Ranjbar et al., 2014) and complete nrDNA sequences (18S, ITS1, 5.8S, ITS2, and 26S regions; Duan et al., 2021); **C,** unresolved (Sanderson and Wojciechowski, 1996; Duan et al., 2016); **D,** Caraganeae is sister to the Chesneyeae + Hedysareae clade, which is revealed in this study based on the complete nrDNA sequences (**ETS**, 18S, ITS1, 5.8S, ITS2, and 26S regions).

**
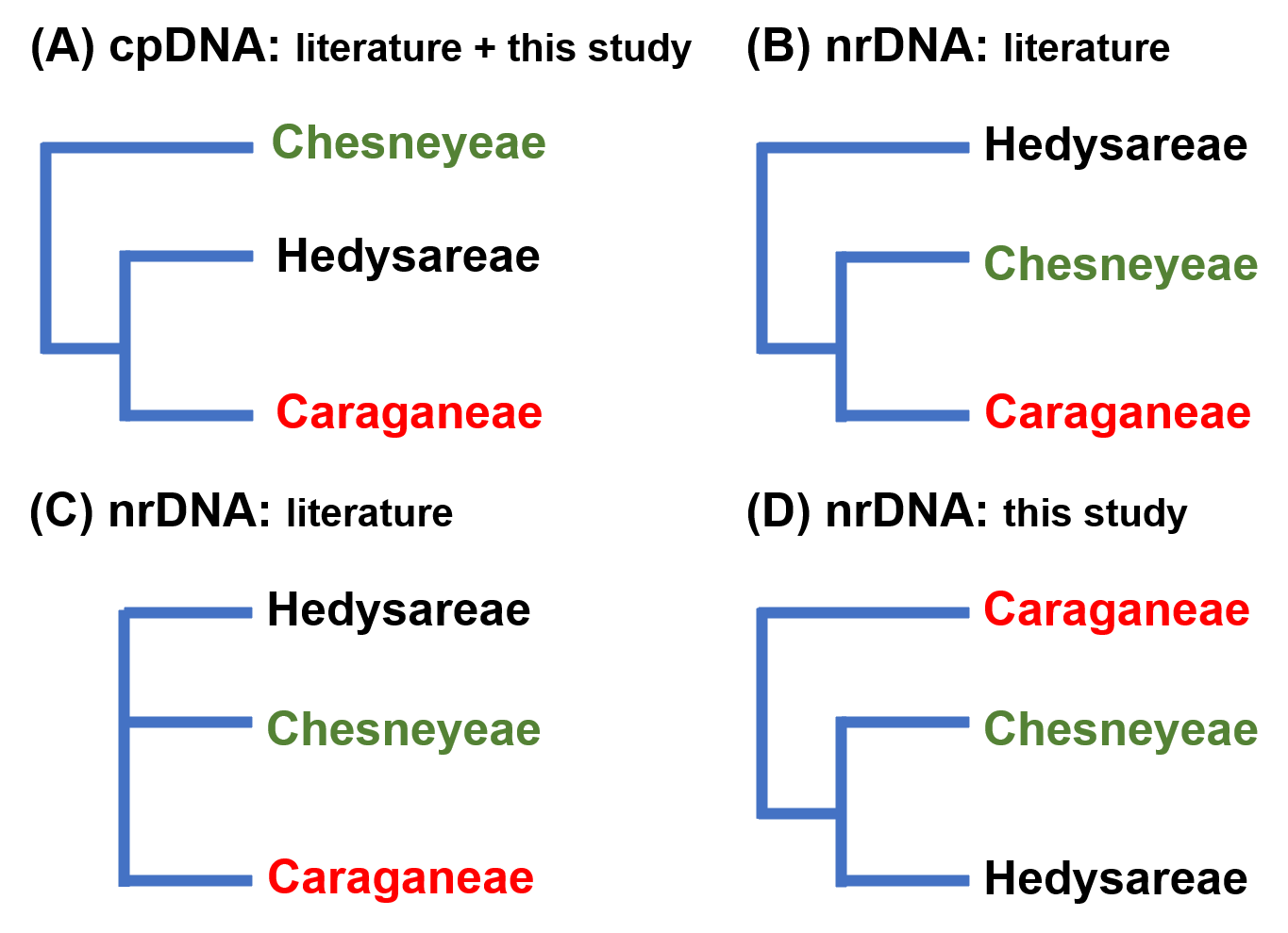
**

**Figure S2.** The Bayesian maximum clade credibility tree of Caraganeae and its related taxa in IRLC based on **chloroplast CDSs**, highlighting the phylogenetic position of Caraganeae. Bayesian posterior probabilities are given above branches, and maximum likelihood bootstrap values are given below branches. Asterisks indicate PP = 1 and LBS = 100%.


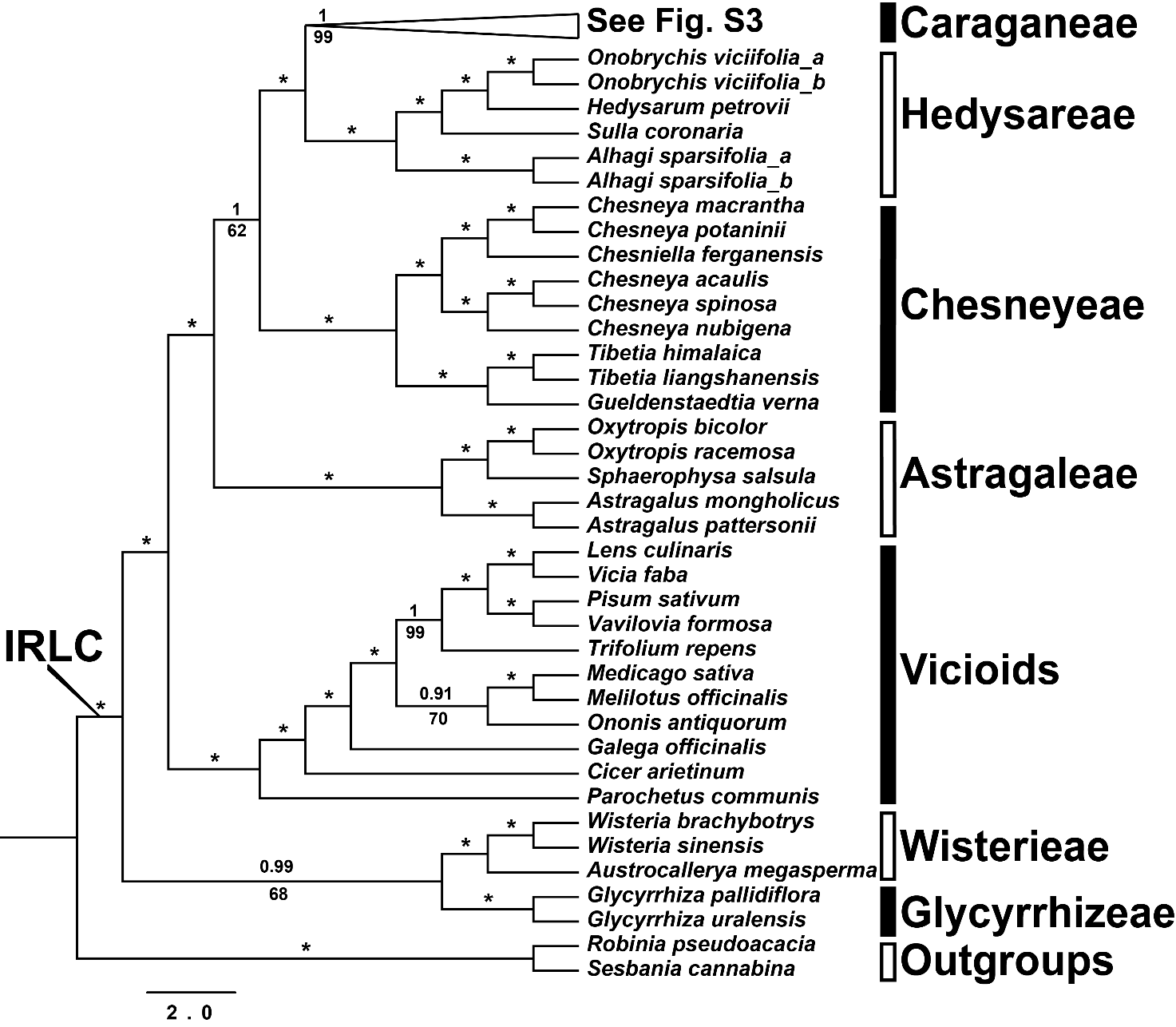


**Figure S3.** The Bayesian maximum clade credibility tree of the tribe Caraganeae based on **chloroplast CDSs**, highlighting the phylogenetic structure within Caraganeae. Bayesian posterior probabilities are given above branches, and maximum likelihood bootstrap values are given below branches. Asterisks indicate PP = 1 and LBS = 100%.


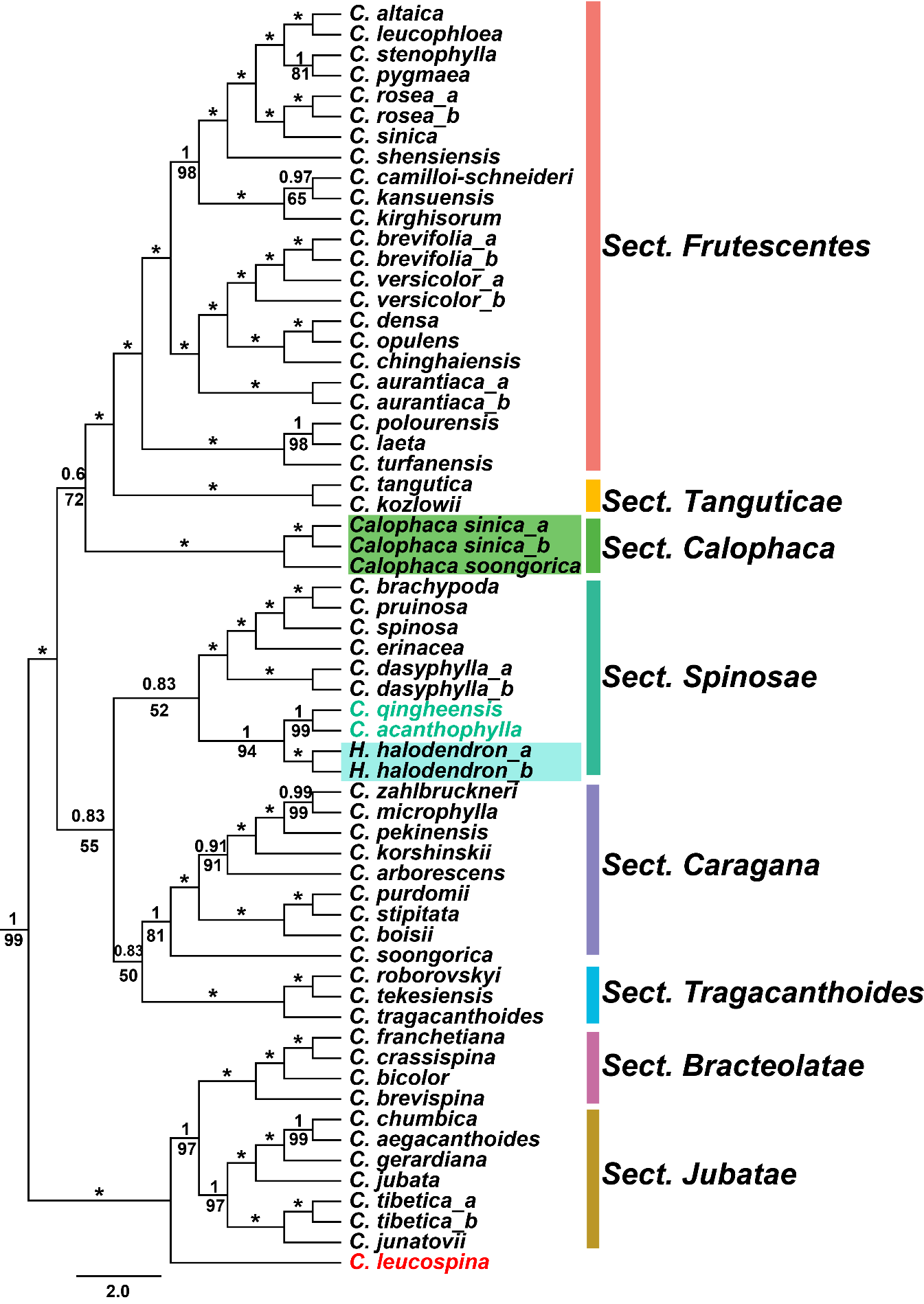


**Figure S4.** Ancestral character reconstruction of Caraganeae, using the Bayesian Binary Method (BBM) implemented in the program RASP. (left) inflorescence: (**A**) One flower per peduncle (solitary or a few in a fascicle); (**B**) Flowers in pairs; (**C**) Umbel with 3-4 flowers; (**D**) Raceme. (right) leaf shape: (**A**) Pseudopalmate; (**B**) Paripinnate; (**C**) Paripinnate on long branchlets and pseudopalmate on short branchlets; (**D**) Imparipinnate.


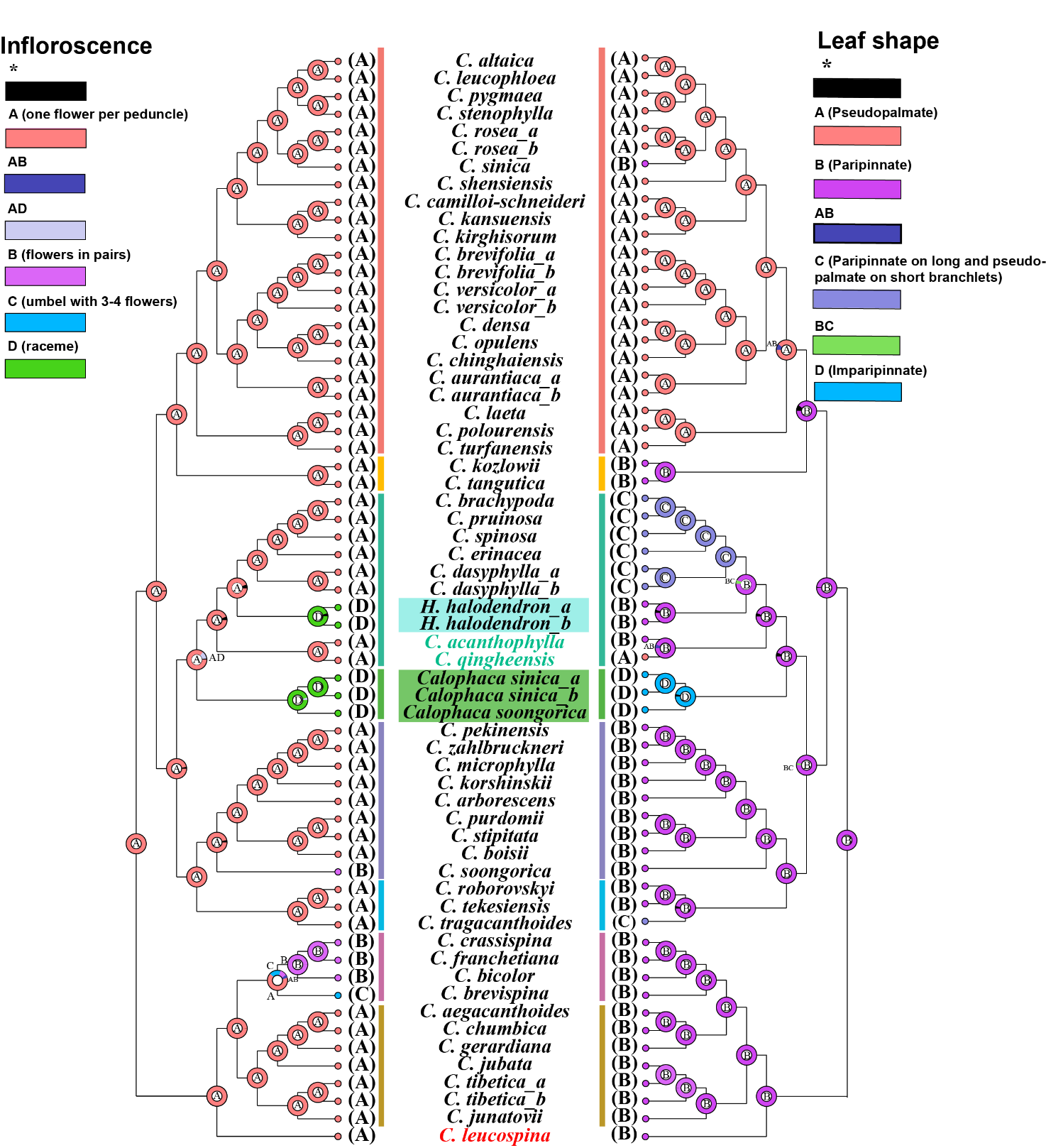


**Figure S5.** The Bayesian maximum clade credibility tree of Caraganeae and its related taxa in IRLC, based on **the nrDNA dataset excluding ETS**. Bayesian posterior probabilities are given above branches, and maximum likelihood bootstrap values are given below branches. Asterisks indicate PP = 1 and LBS = 100%.


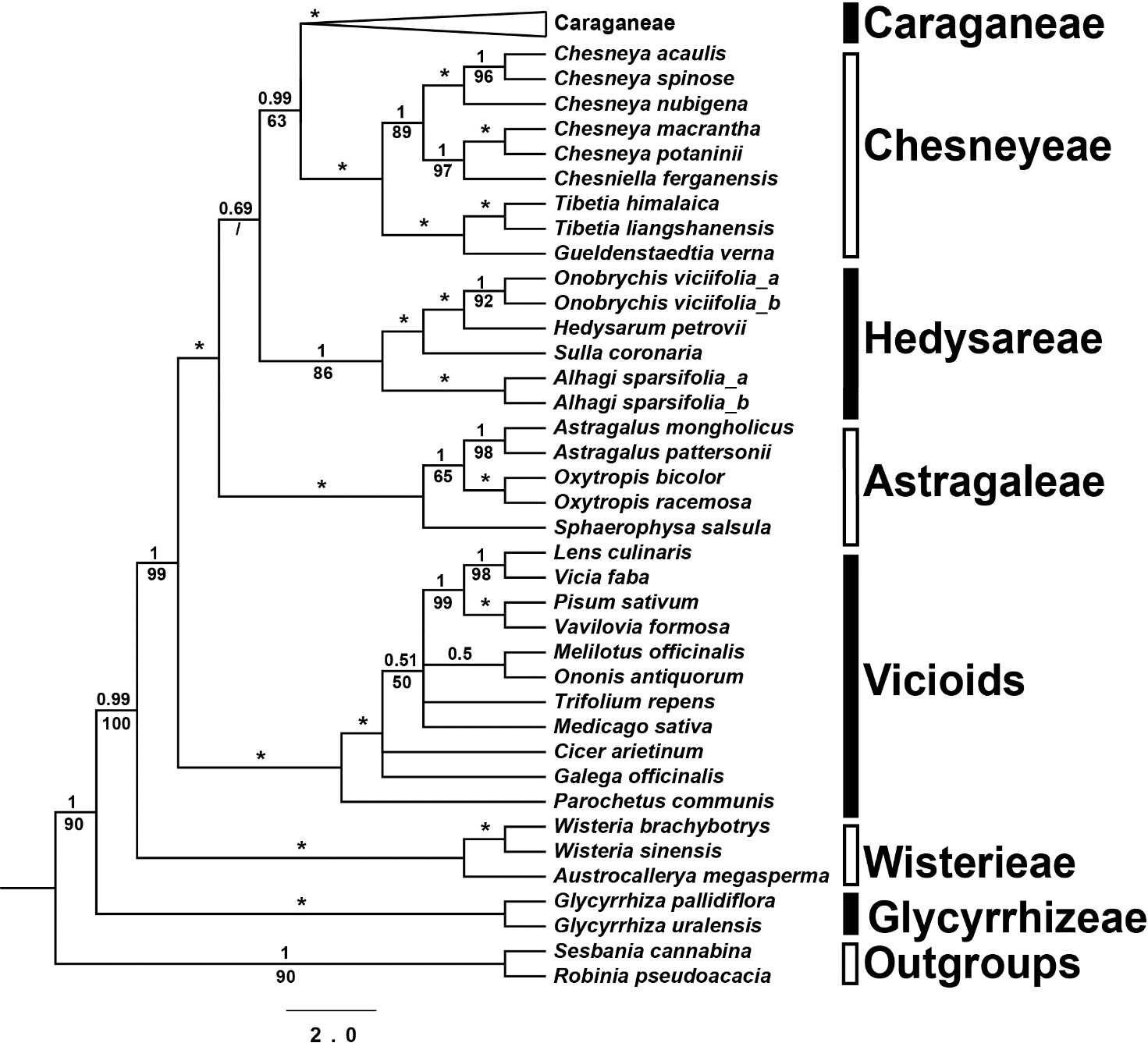

Supplement: Supplementary file 1 — Data S1: ece372638‐sup‐0001‐Supinfo01.docx. [file ECE3-15-e72638-s001.docx]
